# Probing the mechanism by which the retinal G protein transducin activates its biological effector PDE6

**DOI:** 10.1016/j.jbc.2023.105608

**Published:** 2023-12-28

**Authors:** Cody Aplin, Richard A. Cerione

**Affiliations:** 1 Department of Chemistry and Chemical Biology, Cornell University, Ithaca, New York, USA; 2 Department of Molecular Medicine, Cornell University, Ithaca, New York, USA

**Keywords:** signal transduction, structural biology, cryoelectron microscopy, G protein, phosphodiesterase, phototransduction

## Abstract

Phototransduction in retinal rods occurs when the G protein–coupled photoreceptor rhodopsin triggers the activation of phosphodiesterase 6 (PDE6) by GTP-bound alpha subunits of the G protein transducin (Gα_T_). Recently, we presented a cryo-EM structure for a complex between two GTP-bound recombinant Gα_T_ subunits and native PDE6, that included a bivalent antibody bound to the C-terminal ends of Gα_T_ and the inhibitor vardenafil occupying the active sites on the PDEα and PDEβ subunits. We proposed Gα_T_-activated PDE6 by inducing a striking reorientation of the PDEγ subunits away from the catalytic sites. However, questions remained including whether in the absence of the antibody Gα_T_ binds to PDE6 in a similar manner as observed when the antibody is present, does Gα_T_ activate PDE6 by enabling the substrate cGMP to access the catalytic sites, and how does the lipid membrane enhance PDE6 activation? Here, we demonstrate that 2:1 Gα_T_–PDE6 complexes form with either recombinant or retinal Gα_T_ in the absence of the Gα_T_ antibody. We show that Gα_T_ binding is not necessary for cGMP nor competitive inhibitors to access the active sites; instead, occupancy of the substrate binding sites enables Gα_T_ to bind and reposition the PDE6γ subunits to promote catalytic activity. Moreover, we demonstrate by reconstituting Gα_T_-stimulated PDE6 activity in lipid bilayer nanodiscs that the membrane-induced enhancement results from an increase in the apparent binding affinity of Gα_T_ for PDE6. These findings provide new insights into how the retinal G protein stimulates rapid catalytic turnover by PDE6 required for dim light vision.

The phototransduction pathway in retinal rod cells is responsible for vision in dim light and represents one of the most highly amplified and extraordinarily sensitive signaling systems in biology (1, 2, 3). The primary components of this pathway are the G protein–coupled receptor (GPCR) rhodopsin (M_r_ ∼37 kDa), the heterotrimeric G protein transducin (Gα_T_, M_r_ ∼39 kDa, Gβ_1_, M_r_ ∼36 kDa, and Gγ_1_, M_r_ ∼7 kDa), and the heterotetrametric cyclic GMP (cGMP) phosphodiesterase-6 (PDE6), the biological effector of transducin. PDE6 consists of two highly similar but not identical subunits (PDE6α and PDE6β, M_r_ ∼100 kDa), each containing a catalytic site and two domains (GAFa and GAFb) that mediate a negative allosteric regulation by cGMP, together with two smaller (identical) subunits (PDE6γ, M_r_ ∼10 kDa) that are the binding sites for GTP-bound Gα_T_ (Gα_T_·GTP). The phototransduction signaling pathway is initiated upon light absorption by the chromophore *cis*-retinal, which is attached *via* a Schiff base linkage to a lysine residue on the seventh transmembrane helix of opsin, the protein backbone of rhodopsin. The absorption of a single photon by rhodopsin catalyzes multiple cycles of transducin activation. Further amplification occurs upon the activation of PDE6 by Gα_T_·GTP, resulting in hydrolysis of more than 10^3^ cGMP molecules per second. The reduction in cGMP levels closes cGMP-gated cation channels in retinal rod membranes, resulting in membrane hyperpolarization that initiates the signal transmitted to the optic nerve.

The regulation of PDE6 by activated transducin is a critical and unique feature of the phototransduction pathway that enables vision in dim light. PDE6 is a 3′,5′-cyclic nucleotide phosphodiesterase and a member of a superfamily of enzymes that play critical roles in compartmentalizing cyclic nucleotide signaling and regulating many physiological processes (4, 5). The PDE superfamily consists of 11 gene families encoding over 100 distinct isozymes, each with a unique tissue distribution and substrate selectivity profile, making PDEs attractive drug targets due to the prospect of high tissue selectivity with limited off-target effects. Indeed, given their widespread role in human biology, it is unsurprising that PDEs have been found to play a key role in several human diseases including studying retinal diseases, cardiovascular disease, cancer, metabolic diseases, and neurodegenerative disorders (5, 6, 7, 8, 9, 10, 11, 12). While the C-terminal catalytic domain is conserved across all PDEs, most members of the PDE superfamily have an N-terminal regulatory domain, which determines the localization and functional diversity of each isozyme. The PDE6 gene family has three isoforms, each with an N-terminal regulatory domain containing tandem GAF domains. PDE6 is the most catalytically active PDE and is the only family member that has coevolved an autoinhibitory PDE6γ subunit, which enables G-protein–stimulated activation and completely prevents basal activity in the absence of Gα_T_. This is a critical feature that ensures an exceedingly high signal-to-noise ratio for the signaling output required for vision in dim light.

While we and others have probed in some detail the molecular basis by which the GPCR rhodopsin activates its G protein signaling partner transducin, and thus have a good picture of how this key step in phototransduction occurs, less is known from a structural and mechanistic perspective regarding how transducin is able to elicit a striking stimulation of PDE6 catalytic activity. Recently, we described a complex that forms between two GTP-bound recombinant Gα_T_ subunits and PDE6 as determined by cryo-EM (Fig. 1*A*) (13). Based on its structure, we proposed a mechanism by which GTP-bound Gα_T_ subunits bind to the PDE6γ subunits of PDE6 and cause a significant change in their positioning relative to the PDE6α and PDE6β subunits. This appeared to provide both increased access to their catalytic sites and enabled a reversal of the inhibitory constraints on the enzymatic activity imposed by the GAF domains of PDE6. However, to form a stable complex between two Gα_T_ subunits per PDE6 molecule, we took advantage of earlier findings that showed how a bivalent antibody targeting the C-terminal end of Gα_T_ significantly increased its affinity for the effector enzyme (14). It was also necessary to occupy the catalytic sites of the PDE6α and PDE6β subunits with vardenafil, a competitive inhibitor of the substrate cGMP. Based on our structural determination of this complex, we proposed a working model for how GTP-bound Gα_T_ subunits activated PDE6. It involved the combined binding of two Gα_T_ subunits per PDE6 molecule that worked in tandem to release the PDE6γ subunits from the catalytic sites of PDE6α and PDE6β and to reposition the PDE6γ subunits in a manner that removed the inhibitory constraints on catalytic activity imposed by the GAF domains. One key finding of these studies was that the recombinant Gα_T_∗ subunits in the Gα_T_∗–PDE6 complex adopt an upside down conformation relative to the presumed location of the plasma membrane based on post-translational lipid modifications (PTMs) on the C-terminal helices of the PDE6α and PDE6β subunits, the positioning of Gα_s_ bound to adenylyl cyclase, and the positioning of Gα_T_ in the rhodopsin–transducin complex (Fig. 1*B*, PDBs: 6R3Q, 6OY9) (13, 15, 16).

**Figure 1:**
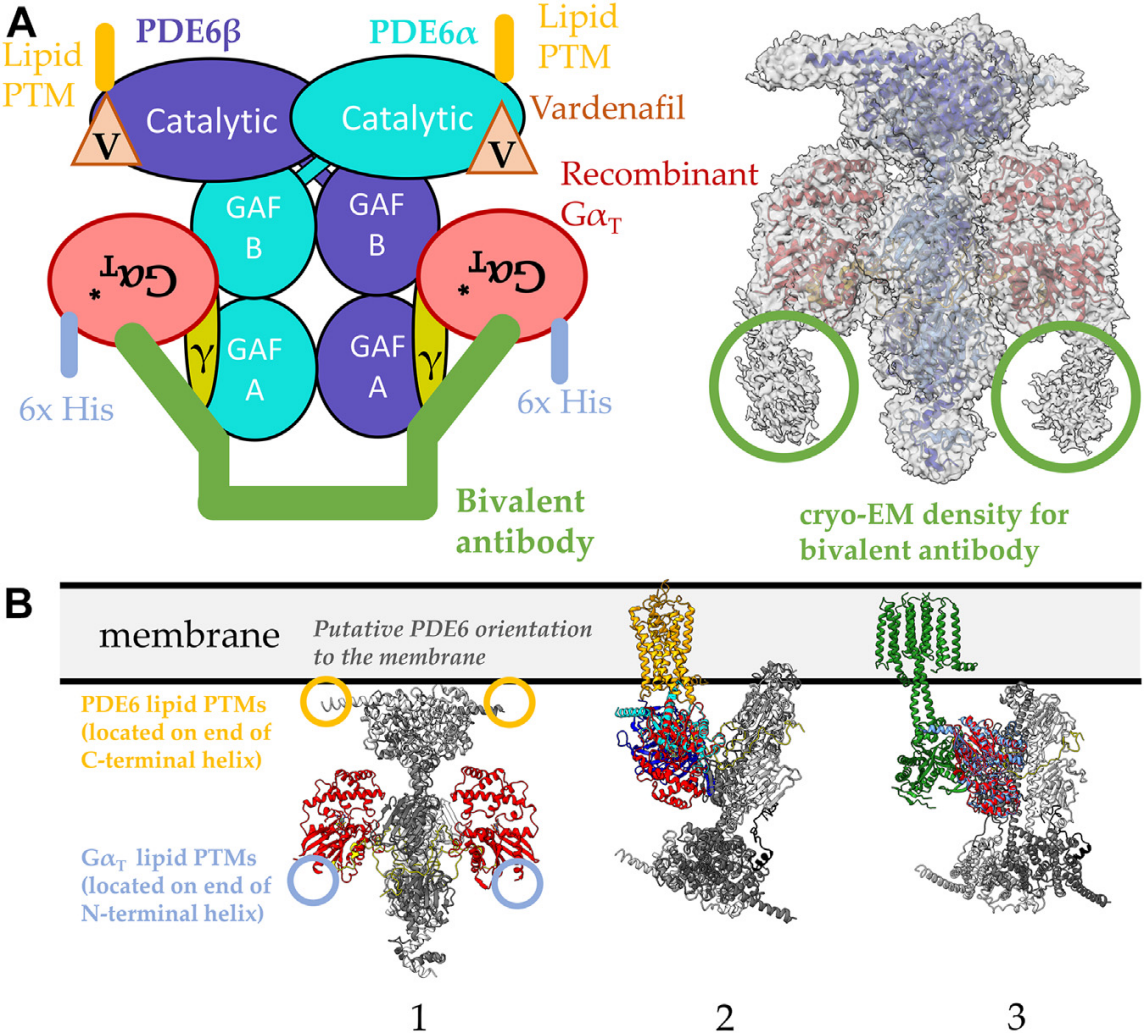
**Depictions of the interactions of GTP-bound transducin Gα subunits with PDE6.***A*, *left:* the cryo-EM structure of the Gα_T_∗–PDE6 complex solved by Gao *et al.* was engineered using recombinant Gα_T_∗ subunits, a bivalent antibody, and the orthosteric inhibitor vardenafil (PDB: 7JSN). *Right:* weak cryo-EM density for the bivalent antibody is observed in the cryo-EM map of Gα_T_∗–PDE6 complex (*red circles*, EMDB: 22,458). *B*, comparison of the orientation of the Gα subunit in different protein complexes. *1*, the presumed association of the 2:1 Gα_T_–PDE6 complex with the membrane, based on the location of lipid modifications on the C-terminal helices of the PDE6α and PDE6β subunits (*yellow*). The *upside-down* orientation of Gα_T_∗ is highlighted by labeling the location of the N-terminal helix of Gα_T_∗, which contains PTMs in native retinal Gα_T_ (*blue*). *2*, orientation of the Gα_T_ subunit in the cryo-EM structure of the rhodopsin–transducin complex (PDB: 6OY9) compared to the Gα_T_–PDE6 complex. One Gα_T_ subunit is shown in the Gα_T_–PDE6 complex for simplicity. The structures are aligned by the Gα_T_ subunits and membrane-bound rhodopsin is used to orient the complexes with the membrane. *3*, orientation of the Gα_s_ subunit in the cryo-EM structure of adenylyl cyclase bound to Gα_s_ (PDB: 6R3Q) compared to the Gα_T_–PDE6 complex. One Gα_T_ subunit is shown in the Gα_T_–PDE6 complex for simplicity. The structures are aligned by the Gα subunits and membrane-bound adenylyl cyclase is used to orient the complexes with the membrane. PDE, phosphodiesterase; PTM, posttranslational lipid modification.

Still, there are many questions regarding this proposed mechanism. They include whether the complex that forms between Gα_T_ and PDE6 in the absence of a bivalent antibody would be the same or different from that observed when the antibody is coupled to Gα_T._ Moreover, is a similar complex assembled when using the native retinal Gα_T_ compared to an *Escherichia coli* recombinant, engineered Gα_T_ subunit, and what is the mechanistic basis for the observations that a membrane environment as provided by retinal rods significantly enhances the ability of Gα_T_ to activate PDE6 (14, 17, 18)? In the studies described below, we have begun to address these questions. We demonstrate that it is possible to form a complex between two recombinant Gα_T_ subunits bound to one PDE6 heterotetramer that appears very similar to the complex formed in the presence of a bivalent antibody targeting Gα_T_. By determining cryo-EM structures for PDE6 bound to the substrate cGMP as well as to various inhibitors, combined with 3D variability analysis (3DVA) to investigate the effects of substrate/inhibitor binding on the PDE6 holoenzyme, we show how occupancy of the catalytic sites helps to loosen their hold on the PDE6γ subunits and enable activated Gα_T_ subunits to reposition them, giving rise to a striking stimulation of enzymatic activity. We present structures of PDE6 bound to either one or two retinal Gα_T_ subunits in the presence of the pan-phosphodiesterase inhibitor 3-isobutyl 1-methylxanthine (IBMX). Finally, using lipid nanodiscs as a membrane mimetic system, we show that the membrane-promoted enhancement of PDE6 activation by Gα_T_ is tightly controlled through the association of Gα_T_ with the membrane and appears to be an outcome of an enhanced affinity of Gα_T_ for the effector enzyme.

## Results

### Examining the ability of Gα_T_ to form a complex with PDE6 in the absence of a bivalent antibody

**Figure 2:**
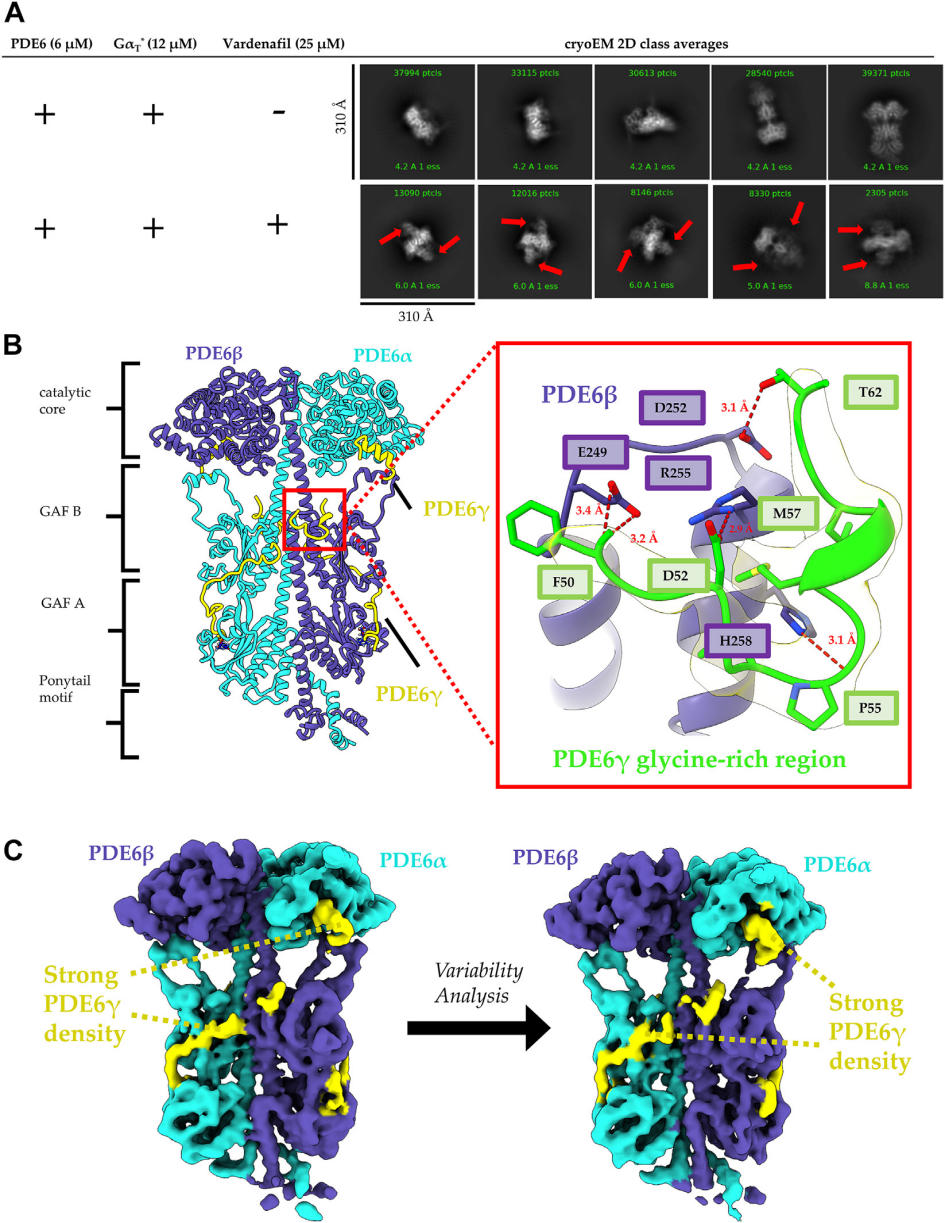
**The glycine-rich region of PDE6γ binds near the PDE6 dimerization interface.***A*, comparison of 2D classes of the Gα_T_∗–PDE6 complex, with and without vardenafil treatment. In the presence of vardenafil, the Gα_T_∗–PDE6 complex forms at a 2:1 stoichiometric ratio and does not readily form in its absence. The Gα_T_∗ subunit is indicated by the red arrows. *B*, the improved cryo-EM structure of the PDE6 heterotetramer. The PDE6α (*cyan*), PDE6β (*purple*), and PDE6γ (*yellow*) are denoted and the domains of the PDE6 holoenzyme are labeled. The hydrogen bonding network between the PDE6γ glycine-rich region (*green*) and PDE6β (*purple*) is shown in the insert (*red*) with the cryo-EM density (*yellow*). *C*, 3DVA shows that PDE6γ remains tightly associated with the PDE6α and PDE6β across all variability components (also see Fig. S3). 3DVA, 3D variability analysis; PDE, phosphodiesterase.

The first high-resolution structure for a Gα_T_–PDE6 complex determined by Gao *et al.* (13) was facilitated by stabilization of the complex with an antibody targeting the C-terminal end of Gα_T_, together with vardenafil, a competitive inhibitor of the substrate cGMP. Here, we set out to determine whether a complex could form between an activated Gα_T_ subunit and PDE6 for structural analysis in the absence of a bivalent antibody and vardenafil. The samples were prepared by preincubating purified native bovine rod PDE6 with a constitutively active recombinant Gα_T_ subunit (designated Gα_T_∗), which was generated by introducing 18 residues from Gα_i_ into Gα_T_ to facilitate expression in *E. coli* and to achieve PDE6 activation, together with two additional mutations (R174C and Q200L) that prevented GTP hydrolysis. After incubating Gα_T_∗ and PDE6 together for 10 min, vardenafil was added to each sample and briefly incubated at room temperature before being applied to the cryo-EM grid. All cryo-EM data acquisition, model statistics, and validation is summarized in Tables 1 and 2. Comparison of the 2D classification images obtained in previous cryo-EM studies of PDE6 to the images generated in this study shows that in the absence of vardenafil, the Gα_T_∗–PDE6 complex is not stably formed. However, upon the addition of vardenafil, the complex forms robustly and the 2D class averages display well-defined structural features with a stoichiometry of two Gα_T_∗ subunits bound per one PDE6 heterotetramer (Fig. 2*A*).

**Table 1:** Cryo-EM data collection, refinement, and validation statistics

Sample	Apo-PDE6 (EMD-42208) (PDB 8UFI)	PDE6-udenafil (EMD-42220) (PDB 8UGB)	PDE6-cGMP (EMD-42234) (PDB 8UGS)	PDE6-IBMX (EMD-42358) (PDB 8ULG)
Data collection and processing				
Magnification	79,000	63,000	63,000	63,000
Voltage (kV)	200	200	200	200
Electron exposure (e−/Å^2^)	50	50	50	50
Defocus range (μm)	−1.2 to −2.4	−1.4 to −2.0	−1.4 to −2.0	−1.0 to −2.6
Physical pixel size (Å)	1.034	1.31	1.31	1.34
Symmetry imposed	C1	C1	C1	C1
Initial particle images (no.)	1,300,728	4,731,596	3,578,031	4,950,970
Final particle images (no.)	457,179	2,050,380	788,557	863,564
Map resolution (Å)	3.1	3.0	3.2	3.2
FSC threshold	0.143	0.143	0.143	0.143
Map resolution range (Å)	2.3–8.8	2.7–8.0	2.8–8.0	2.9–8.0
Refinement				
Initial model used	ModelAngelo	ModelAngelo	ModelAngelo	ModelAngelo
Model resolution (Å)	3.05	2.90	3.01	3.00
FSC threshold	0.143	0.143	0.143	0.143
Map sharpening *B* factor (Å^2^)	−50	−20	−20	−100
Model composition				
Nonhydrogen atoms	14,157	13,726	13,961	14,635
Protein residues	1734	1669	1701	1789
Ligands	6	8	8	8
*B* factors (Å^2^)				
Protein	132.29	103.80	96.21	101.29
Ligand	135.64	108.68	95.71	90.13
RMSD				
Bond lengths (Å)	0.004	0.004	0.002	0.002
Bond angles (°)	0.533	0.629	0.528	0.536
Validation				
MolProbity score	1.59	1.92	1.52	1.55
Clash score	7	7	5	5
Poor rotamers (%)	1.5	1.4	2.0	1.8
Ramachandran plot				
Favored (%)	96.27	94.52	96.63	96.29
Allowed (%)	3.49	5.18	3.32	3.66
Disallowed (%)	0.30	0.30	0.06	0.06

Abbreviation: FSC, Fourier shell correlation.

**Table 2:** Cryo-EM data collection, refinement, and validation statistics for anisotropic maps without a deposited atomic model

Sample	PDE6 bound to two chimera Gα_T_∗ without a stabilizing antibody (EMD-42238)	PDE6 bound to one retinal Gα_T_·GTPγS (EMD-42235)	PDE6 bound to two retinal Gα_T_·GTPγS (EMD-42237)
Data collection and processing			
Magnification	79,000	63,000	63,000
Voltage (kV)	200	200	200
Electron exposure (e−/Å^2^)	50	50	50
Defocus range (μm)	−1.2 to −2.4	−1.0 to −2.6	−1.0 to −2.6
Physical pixel size (Å)	1.034	1.34	1.34
Symmetry imposed	C1	C1	C1
Initial particle images (no.)	552,156	4,950,970	4,950,970
Final particle images (no.)	198,342	59,607	42,479
Map resolution (Å)	4.44	4.15	4.24
FSC threshold	0.143	0.143	0.143

Abbreviation: FSC, Fourier shell correlation.

High-resolution 3D reconstruction of the antibody-free complex was hindered by a preferred orientation. Map quality was assessed using both gold-standard Fourier shell correlation (FSC) analysis as well as 3DFSC (19, 20) for evaluating the directional resolution in the presence of a preferred orientation resulting in map anisotropy. The reported reconstruction resolution was 4.43 Å using the gold-standard FSC resolution (FSC cut-off = 0.143); however, 3DFSC reports a sphericity of 0.830 and a global resolution of 3.3 Å due to missing orientational views in the z-direction (Fig. S1). Despite this, the map displays cryo-EM density for two Gα_T_∗ subunits, allowing for a clear determination of the stoichiometry of the complex (Fig. S1*A*). These results indicate that the ability of activated Gα_T_∗ to form a stable complex with PDE6 requires occupancy of the substrate-binding sites, in this case by a competitive inhibitor. They also show that the induction of a significant repositioning of the PDE6γ subunits by Gα_T_∗ along the PDE6α/PDE6β core is not a unique consequence of the bivalent antibody but rather is a natural outcome of the binding of activated Gα_T_∗ to PDE6.

### Examining the relationship between Gα_T_ binding to PDE6 and occupancy of its catalytic site by a competitive inhibitor of cGMP

We previously assumed that a key role for activated Gα_T_ in stimulating PDE6 activity involved its ability to alter the positioning of the PDE6γ subunits relative to the active sites on PDE6α and PDE6β, thus enabling the substrate cGMP to bind and undergo catalytic turnover. However, because we find that the binding of the competitive inhibitor vardenafil to the catalytic sites of PDE6 is necessary to form a complex between activated Gα_T_ and PDE6, it appears that the reverse is in fact true, that is, occupancy of the enzyme active sites is necessary for a Gα_T_ subunit to bind PDE6. We examined this possibility further using the vardenafil analog udenafil. To see how this inhibitor affects the structural features of PDE6, we first determined a high-resolution reconstruction of the cryo-EM structure for the PDE6 holoenzyme alone (Table 1). The resulting 3.1 Å cryo-EM map shows that the overall structure of the PDE6 heterotetramer is in good agreement with the cryo-EM structure of the PDE6 holoenzyme determined by Gulati *et al.* (PDB: 6MZB, EMDB: 9297) (21) with an overall RMSD of 1 Å, although the resolution was improved from 3.4 Å to 3.1 Å and local resolution peaks were improved from 3.2 Å to 2.3 Å (Table 1). By combining the improved cryo-EM map with ModelAngelo, a newly developed graph neural network-based approach for automated model building (22), we were able to assign residues to a previously unbuilt region of the PDE6γ subunit and present a more complete structural model of the PDE6 holoenzyme. Strong cryo-EM density for residues Pro55 to Met57 allowed for the identification of a new hydrogen bonding network between the glycine-rich region of PDE6γ (residues 55–62) and the dimerization interface of the adjacent PDE6 catalytic subunit (Fig. 2*B*). PDE6γ residues Phe50, Asp52, Pro55, and Thr62 form hydrogen bonds with PDEβ residues Glu249, Arg255, His258, and Asp252, respectively, through interactions both between side chains and with the polypeptide backbone.

Taking advantage of recent advances in analyzing continuous conformational motion in cryo-EM data, we performed 3DVA to investigate PDE6 conformational dynamics. 3DVA is a method implemented in cryoSPARC for fitting 3D linear subspace models to single particle cryo-EM data and can be used to explore conformational flexibility and subunit occupancy (23). The mask and particles from the consensus 3.1 Å refinement were used as an input to 3DVA with three components and the results were filtered to 6 Å resolution for visualization (Fig. S2, *A* and *B*). Notably, despite its intrinsically disordered nature, PDE6γ adopts a largely stable conformation and remains tightly associated with the PDE6αβ subunit core across all variability components (Figs. 2*C* and S3). For the PDE6 heterotetramer, the first variability component resolves bending of the PDE6α GAF domains toward the PDE6β catalytic core, which is associated with destabilization of the “ponytail motif” (Fig. S2*C*). The second variability component resolves twisting of the GAF domains around the dimerization helices (*i.e.*, formed by the two large PDE6α and PDE6β catalytic subunits), which is accommodated by movement of the catalytic cores toward the GAF domains, resulting in a slight compaction of the enzyme (Fig. S2*C*). The third variability component resolves bending of the GAF domains away from the catalytic cores of both PDE6α and PDE6β subunits on one face, which is coordinated with movement of the GAF domains toward the catalytic cores of the other face (Fig. S2*C*).

**Figure 3:**
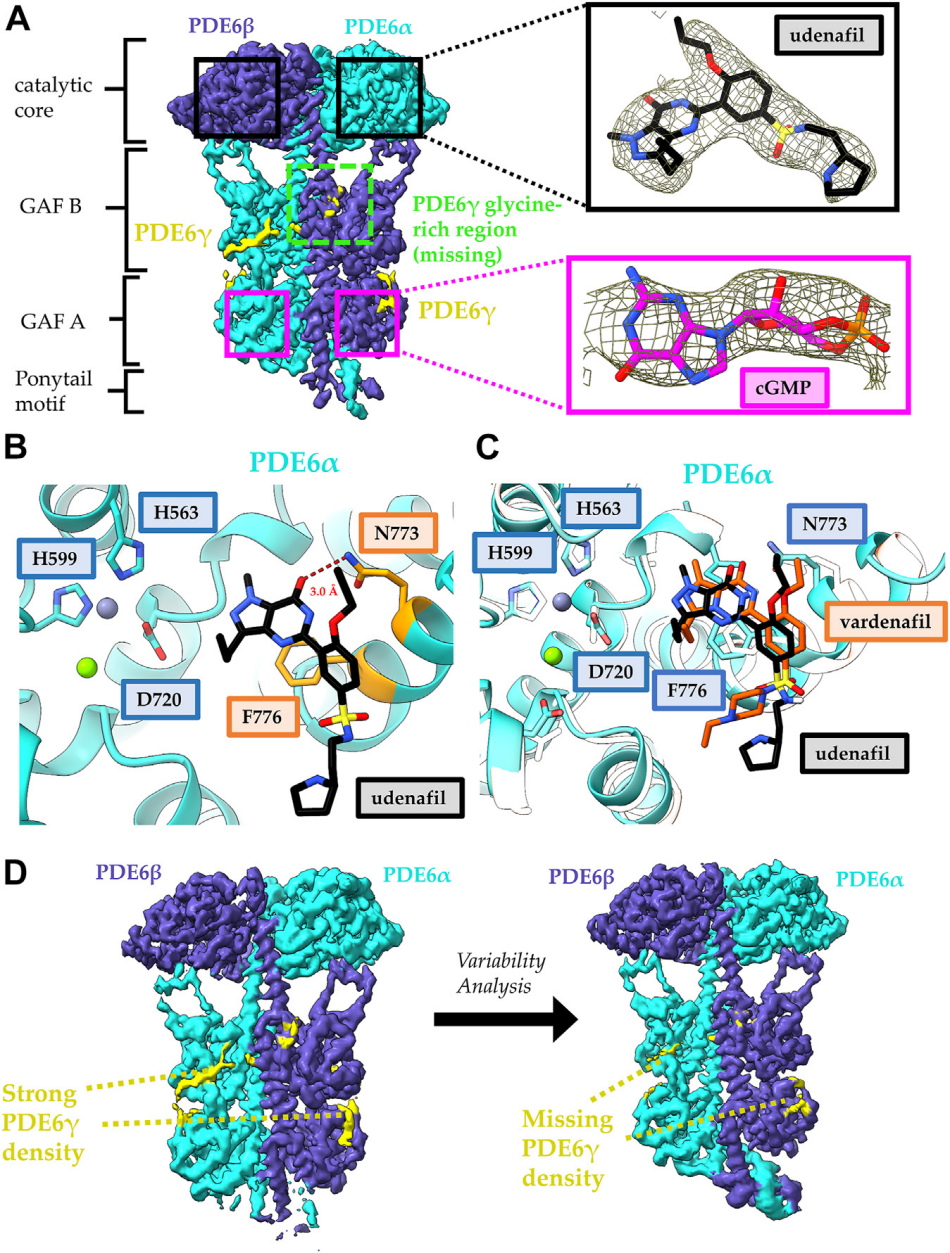
**Bulky, orthosteric PDE inhibitors disrupt PDE6γ binding by sterically competing with the PDE6γ C terminus.***A*, 2.9 Å cryo-EM map of PDE6–udenafil complex. The map density for the PDE6α (*cyan*), PDE6β (*purple*), and PDE6γ (*yellow*) are colored within 4 Å of the atomic model and the location of the udenafil and cGMP binding sites are highlighted with *black and magenta boxes*, respectively. Inset: strong cryo-EM map density is shown for udenafil (*black*) bound at the active site and cGMP (*magenta*) bound to the GAF A domain. The missing density for the PDE6γ glycine-rich region is labeled in a *dashed box* (*green*). *B*, the udenafil (*black*) binding pocket in the catalytic core of PDE6α. Magnesium (*green*) and zinc (*gray*) are shown as *spheres* in the active site and the residues important for metal ion coordination are labeled. The strongest interactions between udenafil and PDE6 are π–π stacking with F776 and hydrogen bonding (*red*) with N773 which are highlighted in *orange*. *C*, comparison of the ligand poses for vardenafil (PDB: 7JSN, *orange*) and udenafil (this paper, *black*) in the PDE6α active site. PDE6α from the udenafil-bound structure is colored *cyan* and PDE6α from the vardenafil-bound structure is colored *white*. Magnesium (*green*) and zinc (*gray*) are shown as *spheres* in the active site and the residues important for metal ion coordination are labeled. *D*, 3DVA analysis of the PDE6–udenafil complex shows a diminished association of PDE6γ with the GAF domains PDE6αβ across all variability components. The map density for the PDE6α (*cyan*), PDE6β (*purple*), and PDE6γ (*yellow*) are colored within 5 Å of the atomic model. 3DVA, 3D variability analysis; cGMP, cyclic GMP; PDE, phosphodiesterase.

We then used cryo-EM to solve a high-resolution structure of PDE6 bound to udenafil (Table 1). The resulting cryo-EM map was refined to 2.9 Å resolution and the initial atomic model was generated using ModelAngelo (Fig. 3). The catalytic cores of the apo- and udenafil-bound PDE6 are remarkably similar, with an overall RMSD of 0.3 Å when aligned by the PDE6α catalytic site; however, there is minimal cryo-EM map density for the C termini of the PDE6γ subunits, which bind near the PDE6 active sites in apo-PDE6, as well as a weakened density for the glycine-rich region of PDE6γ (Fig. 3*A*). The cryo-EM map has local resolution peaks at 2.7 Å for the catalytic cores with strong density for udenafil bound at both active sites on PDE6α and PDE6β and for cGMP bound at their GAF A domains (Fig. 3*A* and Table 1). Udenafil binding is stabilized primarily through π–π stacking with Phe776 and hydrogen bonding with Asn773, in good agreement with structures of PDE5 in complex with other orthosteric inhibitors (Fig. 3*B*). The ligand poses for vardenafil from the structure of the Gα_T_∗–PDE6 complex (PDB: 7JSN) (13) resemble those of udenafil; however, udenafil adopts a slightly more extended conformation (Fig. 3*C*). We then performed 3DVA on the udenafil-bound PDE6 cryo-EM data to compare the conformational dynamics of the inhibitor-bound and apo-states. The mask and particles from the consensus 2.9 Å refinement were used as input to 3DVA with three components and the results were filtered to 6 Å resolution for visualization. Overall, the motions of the udenafil-bound PDE6 complex were similar to what we observe for the apoenzyme. Across all three components, the cryo-EM map density for PDE6γ is diminished while moving along the variability coordinate axis (Figs. 3*D* and S4). This indicates that the binding of udenafil weakens the association of the PDE6γ C terminus with the catalytic sites, due to steric clashes, which would enhance the ability of activated Gα_T_ subunits to bind and reposition the PDE6γ subunits away from the PDE6 catalytic sites.

### Examining the relationship between Gα_T_ binding to PDE6 and occupancy of its catalytic sites by the substrate cGMP

**Figure 4:**
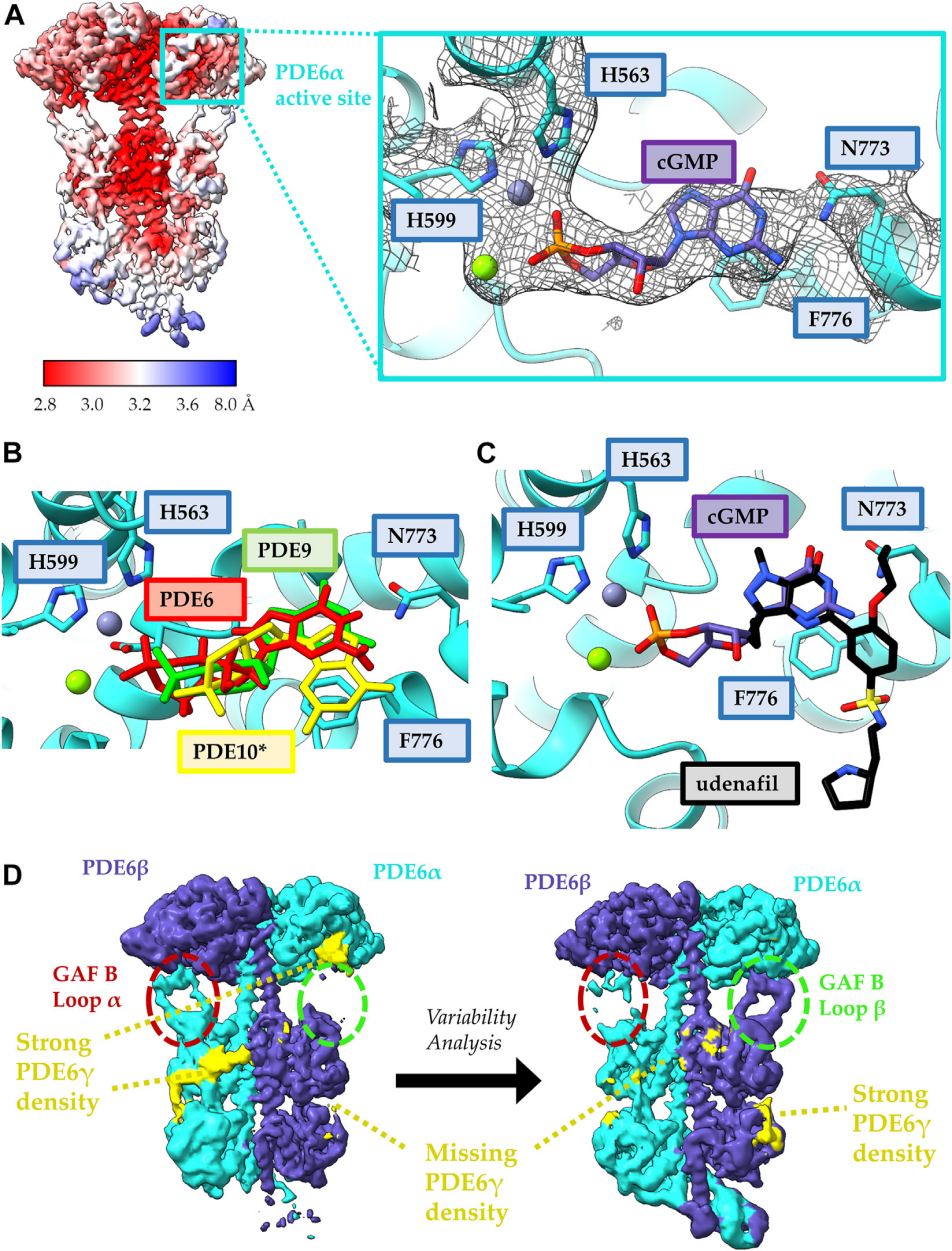
**cGMP binding disrupts PDE6γ binding without a steric clash and without the need for GαT.***A*, local resolution map of the PDE6–cGMP complex, displaying resolution estimates from 2.8 to 8 Å. The inset shows cryo-EM map density for the active site of PDE6α (*cyan*) when cGMP is bound. The density is displayed within 3 Å of the labeled residues. Magnesium (*green*) and zinc (*gray*) are shown as *spheres* in the active site and the residues important for metal ion coordination are labeled. *B*, comparison of the docking of cGMP into PDE6 (*red*), an inhibited PDE9 (*green*), and a catalytically dead PDE10 mutant (*yellow*). The ligand poses for cGMP in PDE6 and PDE9 are very similar; however, the PDE10 mutation exhibits a different docking pose. *C*, comparison of the ligand poses of cGMP and udenafil in the PDE6α (*cyan*) active site. The heterocyclic rings of both ligands are positioned in the same manner for π–π stacking with F776 and form hydrogen bonds with N773. *D*, 3DVA analysis of the PDE6–cGMP complex shows a diminished association between PDE6γ and the PDE6α and PDE6β catalytic subunits across all variability components. The map density for the PDE6α (*cyan*), PDE6β (*purple*), and PDE6γ (*yellow*) are colored within 5 Å of the atomic model. 3DVA, 3D variability analysis; cGMP, cyclic GMP; PDE, phosphodiesteras.

We next attempted to capture the Gα_T_∗–PDE6 complex under catalytic turnover conditions by preparing cryo-EM samples containing PDE6 (3 μM) with the recombinant activated Gα_T_∗ subunit (10 μM) in the presence of saturating amounts of cGMP (5 mM). However, the resulting high-resolution (3.2 Å) reconstruction of the cGMP-bound PDE6 complex showed no cryo-EM map density for Gα_T_∗ and local resolution peaks at ∼2.8 Å (Fig. 4*A* and Table 1). This is likely due to the inability to trap a stable activated Gα_T_∗–PDE6 complex when bound to substrate, as upon catalytic turnover and the dissociation of the product GMP, the affinity of Gα_T_∗ for the enzyme is markedly reduced since the substrate binding site is no longer occupied. Therefore, the only stable complex that we were able to image under saturating conditions of cGMP is PDE6 bound to substrate alone.

The ligand density for the PDE6 active sites shows strong density for cGMP as well as zinc and magnesium ions that are found in other structures of PDE6 (Fig. 4*A*). To our knowledge this represents the first structure solved for retinal PDE6 bound to its substrate. Previous studies using X-ray crystallography were successful in solving structures for cGMP bound to the catalytic domains of phosphodiesterases, either by inhibiting the enzymes through the removal of the divalent metal ions, treating the enzymes with saturating zinc concentrations (PDE9, PDB: 3DYQ and 3DYN), or by introducing a mutation to a conserved aspartic acid residue (D674A) that prevents binding of the zinc ion for catalysis (PDE10A, PDB: 2OUU) (24, 25). The ligand pose for cGMP is highly similar between the PDE9 structures and our PDE6-cGMP structure; however, the pose for the PDE10A D674A mutation is quite different (Fig. 4*B*). This is likely due to the loss of the active site zinc ion in the catalytically dead PDE10A mutant, indicating that this zinc ion is critical for correct positioning of the substrate for catalysis. Like the case for the PDE6–udenafil complex, the guanine ring of cGMP forms hydrogen bonds with N774 and undergoes π–π stacking with Phe776. When comparing the ligand pose of the substrate cGMP to that of competitive inhibitors like udenafil bound to PDE6, the bulky substituent group on udenafil extends outward away from the active site (Fig. 4*C*).

**Figure 5:**
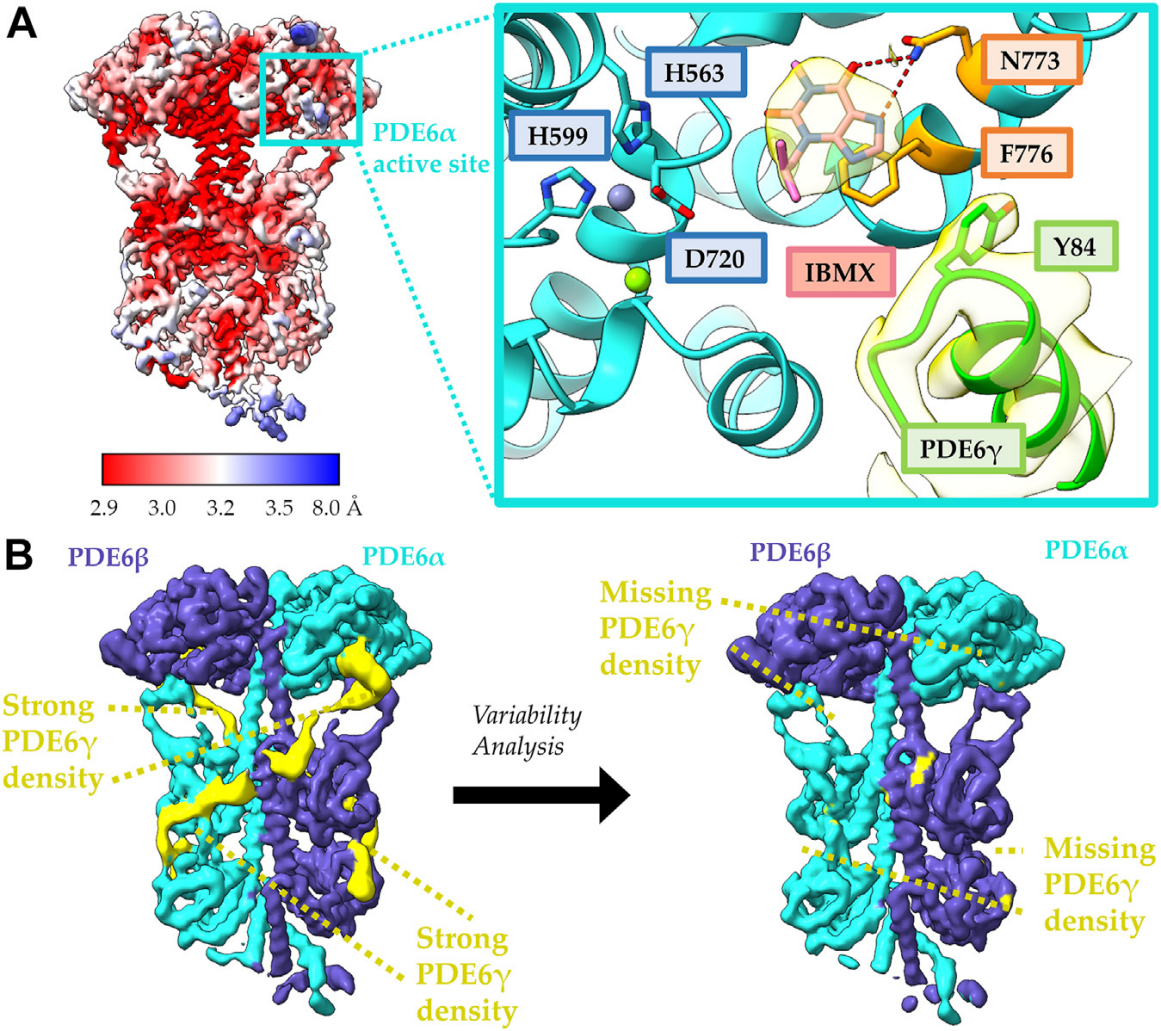
**A small orthosteric inhibitor, IBMX, disrupts PDE6γ binding without a steric clash with the PDE6γ C terminus.***A*, local resolution map of the PDE6–IBMX complex, displaying resolution estimates from 2.9 to 8 Å. The inset shows the IBMX-binding site in the PDE6–IBMX complex. IBMX is stabilized by π–π stacking with F776 and hydrogen bonding (*red*) with N773, which are highlighted in *orange*. Strong cryo-EM density for the PDE6γ subunit and IBMX is shown in *yellow*. Magnesium (*green*) and zinc (*gray*) are shown as *spheres* in the active site and the residues important for metal ion coordination are labeled. *B*, 3DVA analysis of the PDE6–IBMX complex shows a diminished association of PDE6γ with the GAF domains PDE6αβ across all variability components. The map density for the PDE6α (*cyan*), PDE6β (*purple*), and PDE6γ (*yellow*) are colored within 5 Å of the atomic model. 3DVA, 3D variability analysis; IBMX, inhibitor 3-isobutyl 1-methylxanthine; PDE, phosphodiesterase.

Using 3DVA, we investigated the association of PDE6γ with PDE6α and PDE6β in the PDE6–cGMP complex. The overall motions of the PDE6 catalytic subunits within the cGMP–PDE6 complex were similar to those for udenafil bound to the enzyme. Despite the lack of a steric clash with the PDE6γ C terminus, the presence of substrate in the active site again disrupts the association of PDE6γ with the PDE6α and PDE6β subunits (Fig. 5*D*). Across all variability components, as well as the consensus cryo-EM map, the map density for PDE6γ is significantly diminished in comparison to the apo-PDE6 structure. Interestingly, the cGMP bound structure displays greater flexibility in the GAF B loops than that of either apo-PDE6 or the udenafil-bound PDE6 complex. Viewed along variability component 1, the cryo-EM densities for the GAF B loops of PDE6α and PDE6β disappear simultaneously suggesting an increase in flexibility (Fig. S5). Across the same variability component, the density for PDE6γ disappears in accordance with the destabilized GAF B loops, indicating that the binding of the PDE6γ subunits to the PDE6α and PDE6β catalytic subunits may be coupled to the association of the GAF B loops with the catalytic domain. When viewed along variability component 2, the GAF B loops asymmetrically become flexible, and PDE6γ remains associated with the catalytic subunit that maintains contact with the GAF B loops (Fig. S5). When viewed along variability component 3, the GAF B loops are again asymmetrically flexible, and PDE6γ bound to PDE6α remains associated with the catalytic subunit that maintains contact with the GAF B loop of PDE6β, whereas the PDE6γ subunit bound to PDE6β remains largely dissociated regardless of the position of the GAF B loop (Fig. S5). The asymmetry observed in the GAF domain interactions with the PDEα and PDEβ subunits might be related to what we observed in our original Gα_T_∗-PDE6 structure, which raised the possibility that Gα_T_-stimulated PDE6 occurs in an alternating sequence at the two catalytic sites of the enzyme (13).

### Examining the binding of activated retinal Gα_T_ subunits to PDE6

We next wanted to see whether retinal Gα_T_ binds to PDE6 in a similar manner as recombinant Gα_T_∗, which lacks the N-terminal acylation present on the native Gα_T_ subunit (26). Given that occupancy of the substrate binding sites on PDEα and PDEβ facilitates the ability of activated Gα_T_ to reposition the PDEγ subunits to stimulate catalytic activity, we included the pan-phosphodiesterase IBMX, which is closer in size to the substrate cGMP than the vardenafil class of compounds, to help form the retinal Gα_T_–PDE6 complex. Reconstitution of Gα_T_, Gβγ, and rod outer segment (ROS) membranes containing rhodopsin in the presence of GTPγS was used to generate the activated retinal Gα_T_–GTPγS complex. The predominant species, which was resolved to 3.2 Å, lacked detectable density for bound retinal Gα_T_ (Fig. 5*A*). The high-resolution cryo-EM map for the PDE6–IBMX complex has strong density for both IBMX and PDE6γ bound at the catalytic cores, indicating that the two can simultaneously bind to the PDE6α and PDEβ subunits without experiencing the steric clash observed in the PDE6–udenafil complex (Fig. 5*A* and Table 1). IBMX is stabilized in the active site through π–π stacking with Phe776 and hydrogen bonding with Asn773 and assumes a similar conformation to IBMX bound to other PDE catalytic domains (Fig. 5*A*).

We also compared the 3DVA analysis of IBMX bound to PDE6 to that of the apo-PDE6, and to the udenafil-, and cGMP-bound PDE6 structures and found that the overall motions of the enzyme were similar in all cases. Despite the lack of a steric clash with the PDE6γ C terminus, the presence of an orthosteric ligand disrupts the association of PDE6γ with the PDE6α and PDE6β subunits (Fig. 5*B*). Viewed along variability component 1, the cryo-EM density for PDE6γ is symmetrically disrupted in the IBMX-bound complex, disappearing from both the PDE6α and PDE6β subunits at the same time (Fig. S6). The ponytail region appears to be more stable than in the apo-PDE6 structure, and the density for PDE6γ is fully connected from the PDE6α/PDE6β catalytic core to the glycine-rich region bound at the dimerization interface. This allowed for the modeling of an additional stretch of PDE6γ that had been unmodeled in previous structures due to its flexibility. When viewed along variability component 2, the density for the PDE6γ subunit bound to the PDE6α subunit remains associated with both the catalytic and GAF domains; however, the PDE6γ subunit bound to the PDE6β subunit completely dissociates from the heterotetramer (Fig. S6). This analysis supports the notion that a cGMP competitive inhibitor, like the substrate cGMP, is helping to drive the dissociation of the PDE6γ subunits from the catalytic sites of PDE6 even in the absence of a steric clash with the PDE6γ C terminus.

**Figure 6:**
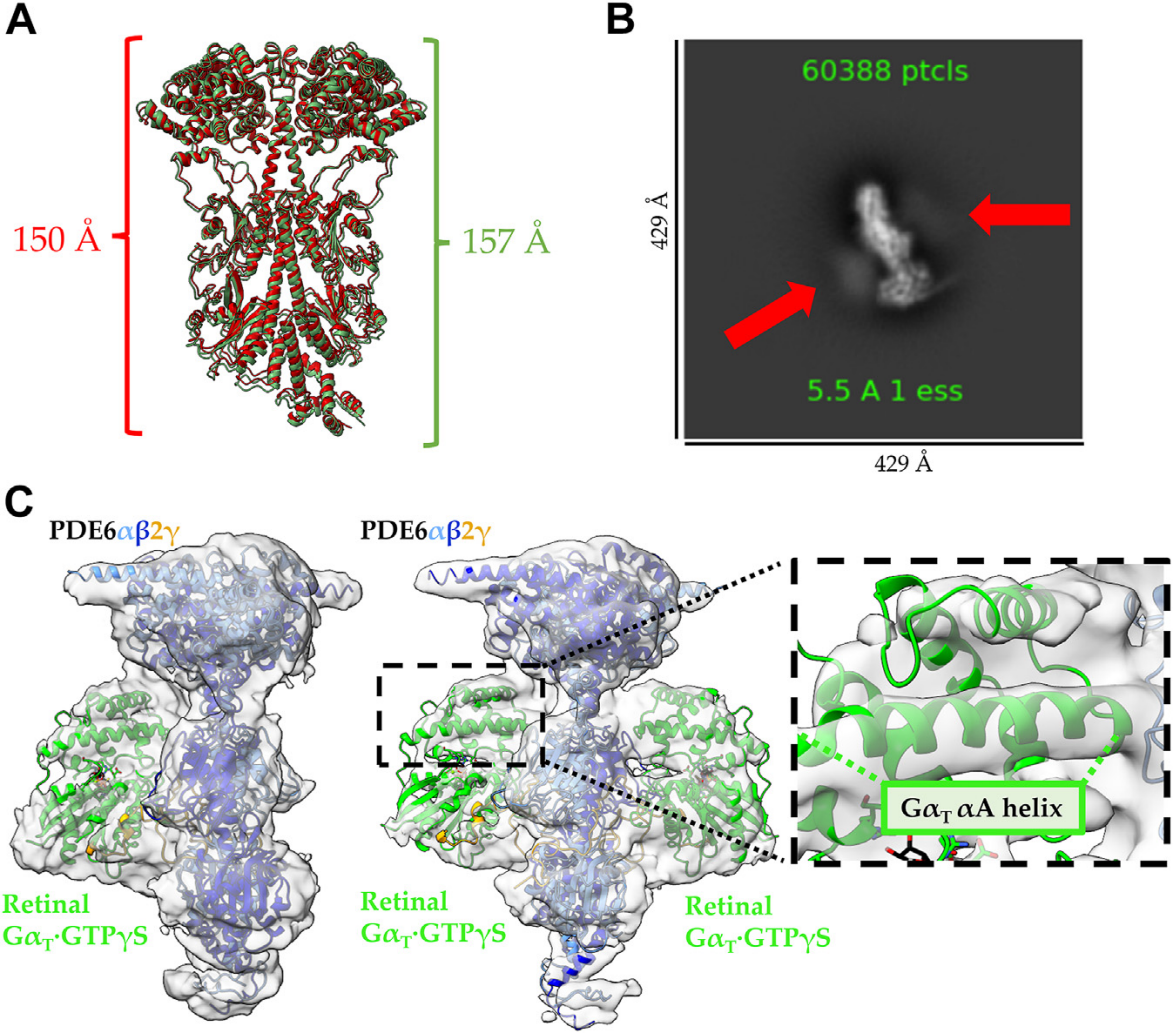
**Retinal GαT forms a PDE6-GαT complex with both 1:1 and 1:2 stoichiometric ratios in the presence of IBMX.***A*, IBMX-bound PDE6 (*green*) is elongated compared to apo-PDE6 (*red*). The distance was measured between residues Glu8 and Glu510 of the PDE6β subunit. *B*, 2D class of IBMX-bound PDE6 in the presence of retinal Gα_T_·GTPγS. Complex formation is diminished in comparison to vardenafil treatment; however, weak density for Gα_T_ is observed (*red arrows*). *C*, low-resolution cryo-EM maps of the retinal Gα_T_·GTPγS–PDE6 complex with both 1:1 and 1:2 stoichiometries. The cryo-EM map density for Gα_T_·GTPγS is strong for the αA helix of Gα_T_ allowing for the determination of its upside-down orientation relative to PDE6 (see Figs. 1*B* and S5). IBMX, inhibitor 3-isobutyl 1-methylxanthine; PDE, phosphodiesterase.

The overall architecture of the PDE6–IBMX complex resembles the apo-PDE6 structure; however, a slight elongation of the holoenzyme results in an RMSD of 1.3 Å. (Fig. 6*A*). This elongation was also observed in the Gα_T_∗–PDE6 complex (PDB: 7JSN) (13), indicating that it may be important for the recruitment of Gα_T_ as opposed to arising upon Gα_T_ binding. Upon careful inspection, weak density for Gα_T_ was observed in some of the 2D class averages (Fig. 6*B*), whereas Iterative rounds of *ab initio* reconstruction and heterogeneous refinement of the complexes using cryoSPARC revealed two subpopulations of PDE6 bound to one or two retinal Gα_T_·GTPγS subunits in the PDE6-IBMX dataset (Fig. 6*C*). High-resolution reconstruction of both complexes was limited due to map anisotropy and a lack of particles (Table 2). Map quality was assessed using gold-standard FSC and 3DFSC methods. For the 2:1 retinal Gα_T_·GTPγS–PDE6 complex, the reported reconstruction resolution using just 42,479 particles (less than 1% of initial particle picks) was 4.24 Å based on the gold-standard FSC resolution (FSC cut-off = 0.143); however, 3DFSC yields a sphericity of 0.747 and a global resolution of 6.99 Å due to missing orientational views in the *x*-direction (Fig. S7, *A*–*C*). For the 1:1 retinal Gα_T_·GTPγS–PDE6 complex, the reported reconstruction resolution obtained for 59,607 particles (approximately 1% of initial particle picks) was 4.15 Å using the gold-standard FSC resolution (FSC cut-off = 0.143); whereas 3DFSC reports a sphericity of 0.760 and a global resolution of 6.58 Å due to missing orientational views in the *x*-direction (Fig. S7, *D*–*F*). These results suggest both maps are highly anisotropic, although the map quality is still sufficient for generating reliable docking models of the structures of both Gα_T_–PDE6 complexes given the existence of previously solved structures of the individual protein components. To generate a model of the retinal Gα_T_·GTPγS–PDE6 complexes, the crystal structure of retinal Gα_T_ bound to GTPγS (PDB: 1TND) was aligned to and substituted for the Gα_T_∗ subunits from the atomic model of the chimeric Gα_T_∗–PDE6 complex from Gao *et al.* (PDB: 7JSN) (27). Importantly, the positioning of both retinal Gα_T_·GTPγS subunits is in excellent agreement with the positioning of the GTP-bound Gα_T_∗ subunits in the 2:1 complex solved by Gao *et al.* (13), indicating that a similar complex forms with the natively purified retinal Gα_T_ that has an N-terminal acylation (*e.g.*, myristylation and/or laurylation) (26). The cryo-EM maps display modest density for the retinal Gα_T_·GTPγS subunit. However strong density for the helical domain, particularly around the αA helix, permitted reliable docking of retinal Gα_T_ (Fig. 6*C*). As observed by Gao *et al.* in the Gα_T_∗–PDE6 complex with the bivalent antibody, the retinal Gα_T_ subunits in both the 2:1 and 1:1 Gα_T_·GTPγS–PDE6 complexes adopt an upside down conformation relative to the presumed location of the plasma membrane based on C-terminal PTMs on the PDE6α and PDE6β subunits and the positioning of Gα_s_ bound to adenylyl cyclase (Fig. S8, PDB: 6R3Q) (13, 15). Taken together, this strongly supports the notion that the inclusion of the bivalent antibody and use of the chimeric Gα_T_∗ subunit did not influence the structure of the Gα_T_–PDE6 complex solved by Gao *et al.* and that in an aqueous solution, the activated Gα_T_ subunits drive PDE6 activation through the displacement of the C terminus of the PDE6γ subunits.

### Investigating how the lipid bilayer enhances Gα_T_-stimulated PDE6 activation

The presence of a membrane bilayer has been shown to provide a significant boost to transducin-stimulated PDE6 activity (14, 17); however, the mechanism by which this occurs has been elusive. In particular, the role of the lipid modifications on the C termini of PDE6α and PDE6β and the N terminus of Gα_T_ remains unclear. To address this question, we used lipid nanodiscs as a membrane mimetic system to investigate the activation of PDE6 by retinal Gα_T_·GTPγS and recombinant 6x-histidine (His)-tagged Gα_T_∗ subunits.

**Figure 7:**
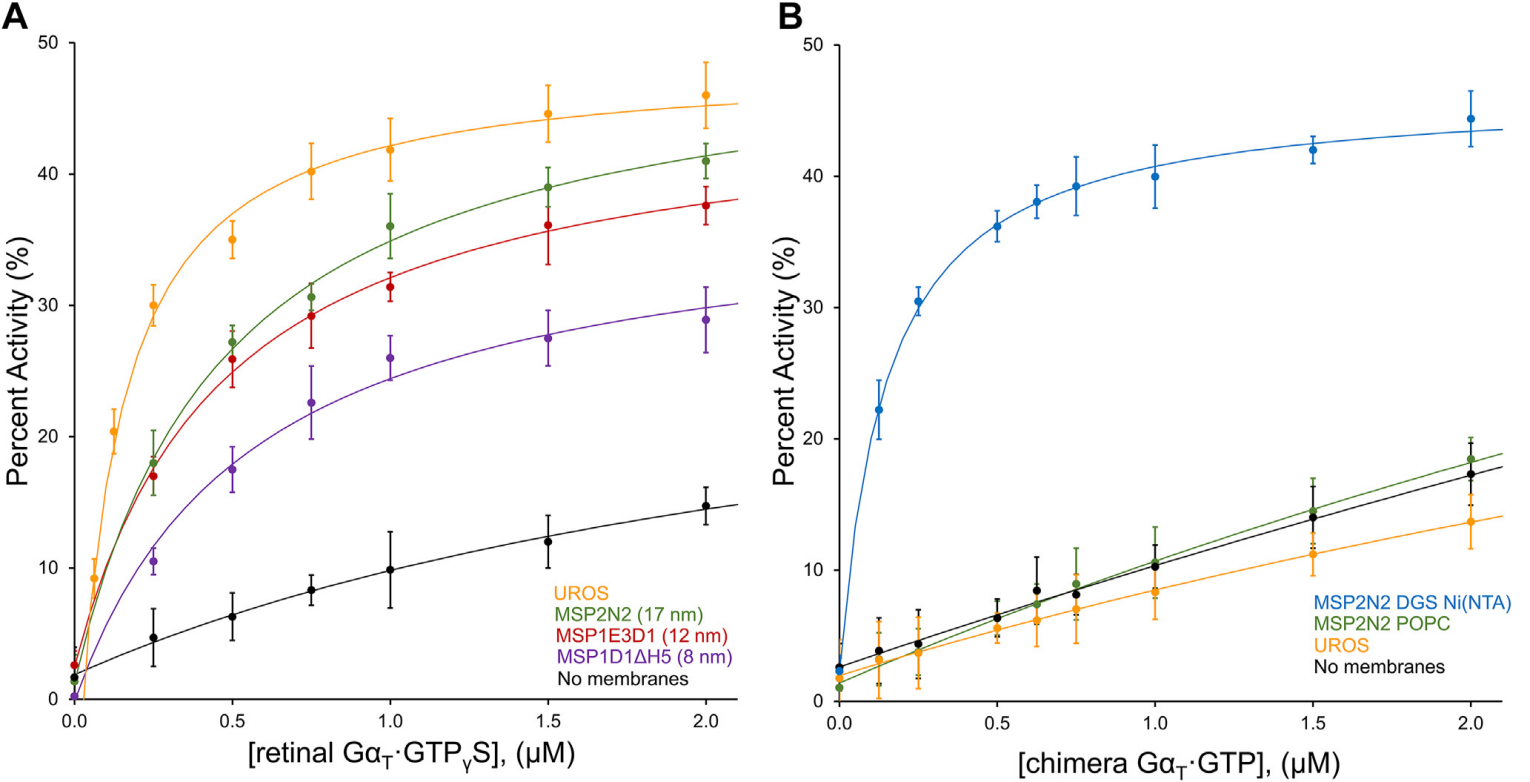
**Transducin-stimulated PDE6 activity in the presence of lipid nanodiscs.***A*, retinal Gα_T_·GTPγS–stimulated PDE6 activity in the presence of UROS (*orange*), MSP2N2 nanodiscs (*green*), MSP1E3D1 nanodiscs (*red*), and MSP1D1Δh5 nanodiscs (*purple*). A control without membranes is included for comparison (*black*). All nanodiscs contain 100% POPC lipids. Data is shown as the average of three independent experiments with the SD shown as error bars (n = 3). *B*, chimera Gα_T_∗-stimulated PDE6 activity in the presence of UROS (*orange*), MSP2N2 POPC nanodiscs (100% POPC, *green*), and MSP2N2 POPC DGS Ni(NTA) nanodiscs (95% POPC, 5% DGS Ni(NTA), *blue*). A control without membranes is included for comparison (*black*). Data is shown as the average of three independent experiments with the SD shown as error bars (n = 3). PDE, phosphodiesterase; POPC, 1-palmitoyl-2-oleoyl-glycero-3-phosphocholine.

We first generated nanodiscs ranging in size from 8 nm (MSP1D1Δh5) to 17 nm (MSP2N2) containing a lipid bilayer of 1-palmitoyl-2-oleoyl-glycero-3-phosphocholine (POPC) and assayed their ability to provide a membrane-induced enhancement of retinal Gα_T_·GTPγS-stimulated PDE6 activity. We found that while each of the nanodiscs provided a boost to PDE6 activity, none of them fully captured the stimulation that is observed with the native rod outer segment membranes (UROS) (Fig. 7*A*). Each of the nanodisc constructs enabled a maximal activity that approached the native UROS membranes. While the apparent affinity of the Gα_T_–PDE6 interaction was somewhat diminished in the nanodisc systems compared to UROS membranes, it appeared to be significantly higher relative to when assaying complex formation in the absence of lipids. We then tested the ability of the recombinant His-tagged Gα_T_∗ to stimulate PDE6 activity in the presence of the nanodiscs and UROS membranes. In the absence of the native lipid modification on Gα_T_∗, we observed no membrane stimulated activity using UROS or MSP2N2 POPC nanodiscs despite the presence of the native lipid modifications on the C termini of the PDE6α and PDE6β subunits (Fig. 7*B*). To attempt to restore the membrane-stimulated enhancement in PDE6 activation when using His-tagged Gα_T_∗, we generated nanodiscs with a functionalized lipid bilayer containing 95% POPC lipids and 5% 1,2-dioleoyl-sn-glycero-3-[(N-(5-amino-1-carboxypentyl)iminodiacetic acid)succinyl] (nickel salt) (DGS Ni(NTA)) lipids. The Ni(NTA) headgroup on the DGS lipids is able to conjugate a 6x-His tag with very high affinity, estimated to be in the picomolar range. Using the recombinant His-tagged Gα_T_∗, we found that MSP2N2 nanodiscs containing 5% DGS Ni(NTA) lipids can achieve maximal membrane-stimulated activation of PDE6 as observed with retinal Gα_T_·GTPγS in UROS membranes. In addition, the apparent affinity of the Gα_T_∗–PDE6 interaction is nearly identical to the native UROS membranes, suggesting that the inclusion of the DGS Ni(NTA) enables the 6x-His tag on the recombinant Gα_T_∗ to mimic the lipid modification on natively purified retinal Gα_T_. These results suggest that the observation of membrane-stimulated PDE6 activity is tightly controlled by the association of Gα_T_ with the lipid bilayer and suggests that the UROS membranes provide a membrane stimulated boost that exceeds that of POPC lipids due to an increased association of retinal Gα_T_ for the UROS membranes and an enhanced binding to PDE6.

## Discussion

**Figure 8:**
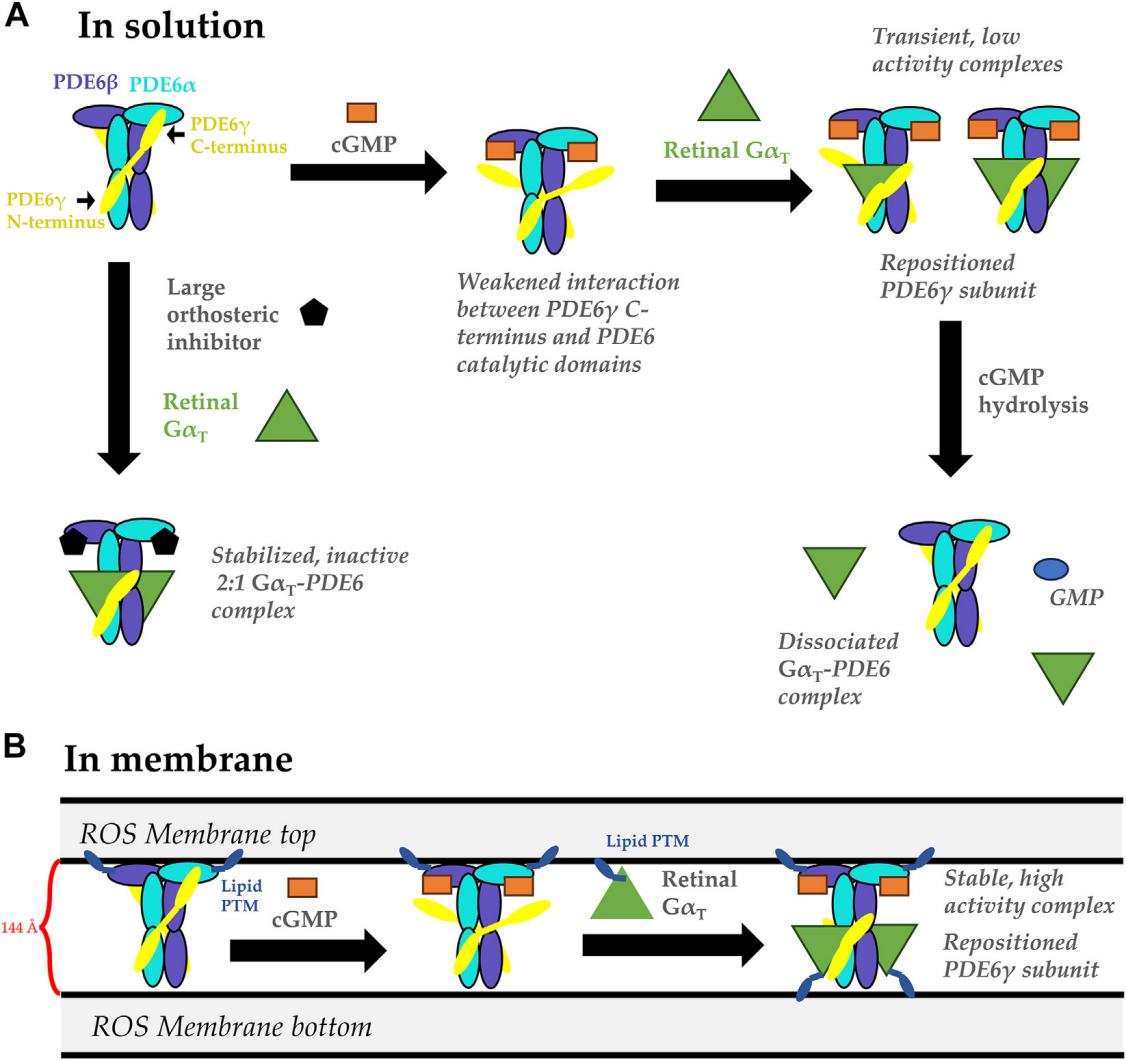
**Activation mechanism of PDE6 by Gα**_**T**_**.***A*, in a solution environment, cGMP is able to bind at the PDE6 catalytic sites, loosening the binding of PDE6γ. This promotes the recruitment of Gα_T_ by the PDE6γ C terminus, resulting in the formation of transient, low activity Gα_T_–PDE6 complexes with both 1:1 and 2:1 stoichiometric ratios. These complexes dissociate after hydrolysis. In the presence of large orthosteric inhibitors, such as vardenafil, an inactive but stable 2:1 Gα_T_–PDE6 complex forms. *B*, in a membrane environment, retinal Gα_T_ subunits use lipid PTM to anchor to the adjacent membrane promoting the formation of high-affinity, stable Gα_T_–PDE6 complexes with displaced PDE6γ subunits, driving maximal PDE6 catalytic activity. cGMP, cyclic GMP; PDE, phosphodiesterase; PTM, posttranslational lipid modification.

Many GPCR pathways, including those responsible for cardiovascular function, neurotransmission, and sensory perception, use cyclic nucleotides such as cAMP and cGMP as second messengers. Upon GPCR catalyzed GDP-GTP exchange on a G protein signaling partner, GTP-bound Gα subunits are generated, which interact directly with nucleotidyl cyclases to control the intracellular concentration of cyclic nucleotides by stimulating or inhibiting their production. The phototransduction system in retinal rods represents a unique example of a GPCR signaling pathway that provides a highly amplified output to enable vision under conditions of dim light. Previous structural studies using both X-ray crystallography and cryo-EM have provided the molecular details by which various GPCRs including the photoreceptor rhodopsin engage their cognate G protein partners to catalyze GDP-GTP exchange reactions that activate the G proteins, priming them for their interactions with biological effector proteins (16, 28, 29, 30, 31, 32, 33, 34, 35). However, to date, much less is known about the underlying mechanisms by which activated G proteins engage and regulate their effectors. Recently, we provided an initial clue for the case of vertebrate vision by solving a 3D cryo-EM structure of two GTP-bound recombinant Gα_T_ subunits bound to one PDE6 heterotetramer (13). However, because this complex consisted of a recombinant, engineered Gα_T_ subunit that was bound to a bivalent antibody, questions arose regarding whether a similar complex would form with native retinal subunits alone (36, 37, 38). Moreover, there have been questions regarding how Gα_T_ subunits promoted PDE6 activation and if this involved altering the positions of their PDEγ binding partners to provide access to catalytic sites on PDE6α/PDE6β for the substrate cGMP. The studies described here are now beginning to provide answers to these questions. Indeed, we have found that similar complexes can form between both recombinant and native retinal Gα_T_ with PDE6 that give rise to a striking repositioning of the PDE6γ subunits along the PDE6α/PDE6β core. Interestingly, this repositioning is not absolutely required for the binding substrate but rather to enable engagement with the GAF domains to remove their inhibitory constraint on catalytic activity. Moreover, occupancy of the catalytic sites by cGMP or competitive inhibitors can help to dislodge the PDE6γ subunits from the PDE6α/PDE6β catalytic core, which enables GTP-bound Gα_T_ subunits to reposition the PDE6γ subunits, driving the formation of a stable Gα_T_–PDE6 complex (Fig. 8*A*).

Our high-resolution structure of PDE6 holoenzyme revealed a detailed interaction network between the glycine-rich region of PDE6γ and the PDE6α/β dimerization interface. This region of PDE6γ primarily consists of residues that have been previously shown to be important for Gα_T_ binding including amino acids 55 to 62, which have been linked to the association of Gα_T_ with the polycationic region of PDE6γ (39, 40, 41). Additionally, the interaction of PDE6γ with PDEβ His258 has been implicated in the pathogenesis of autosomal dominant congenital stationary night blindness, where a H258N mutation plays a causative role (42, 43, 44). Previous studies have shown that PDE6γ is necessary as a cochaperone alongside AIPL1 for the proper folding of PDE6 and suggested that the H258N mutation may disrupt PDE6γ binding to PDEβ, reducing the levels of the functional enzyme, and leading to autosomal dominant congenital stationary night blindness (45). Our high-resolution structure reveals that PDEβ H258 makes a critical hydrogen bond with the peptide backbone of PDE6γ P55. This newly defined interaction network provides strong experimental evidence that each PDE6γ subunit interacts with both PDE6α and PDE6β subunits, suggesting that allosteric regulation of PDE6 by the GAF domains in adjacent catalytic subunits may be facilitated by direct communication through PDE6γ. In addition, the cryo-EM structure of the PDE6–IBMX complex enabled reconstruction of the entire C terminus of PDE6γ from Gly51-Ile86, providing a more complete picture of how this intrinsically disordered protein binds to the catalytic domains of PDE6α/PDE6β. These modeling efforts were facilitated by the use of ModelAngelo, a machine learning approach to automated model building that allowed us to limit the user bias introduced when constructing an atomic model of regions with relatively weak electron density, especially when it is discontinuous with the rest of the protein chain. Importantly, because there are existing structures of the N and C termini of PDE6γ bound to the PDE6αβ subunits, these newly developed models have strong supporting evidence.

The studies described here also provide insights into the structural effect of orthosteric inhibition on the PDE6 heterotetramer. Our high-resolution structures lack cryo-EM density for the PDE6γ C terminus near the PDE6 catalytic core, indicating that when large orthosteric inhibitors such as udenafil (516.7 g/mol) bind to PDE6, the PDE6γ C-terminus clashes with the ligand at the active site but remains bound to the PDE6α and PDE6β subunits through interactions with the dimerization domain and the GAF domains. Previous cryo-EM studies of PDE6 suggested *via* inspection of 2D classes that upon treatment with sildenafil, the PDE6α/β GAF B loops become more flexible, suggesting direct communication between the active site and the regulatory GAF domains (21). We found strong density for the GAF B loops in the consensus cryo-EM structure of udenafil-bound PDE6, which adopts a highly similar conformation to the GAF B loops found in the apo-PDE6 structure (Fig. S9). However, using 3DVA, we found that the density for the GAF B loops is weakened in response to inhibitor binding, supporting the notion of increased flexibility in this region.

We also found that the native substrate, cGMP, as well as the pan-phosphodiesterase inhibitor IBMX, can disrupt PDE6γ binding despite not having any steric clashes with the PDE6γ C terminus. IBMX (222.3 g/mol) is smaller than cGMP (345.2 g/mol) and disrupts PDE6γ binding like the sildenafil class of inhibitors. However, IBMX results in minimal changes to the GAF B loops. In comparison, cGMP binding drives significant flexibility in the GAF B loops of both catalytic subunits. This finding further supports the notion that orthosteric ligand binding can impact the structure of the GAF B loops suggesting that this interaction plays an important role in the allosteric communication between the GAF domains and the catalytic domains of PDE6. Importantly, in the case of all ligands and regardless of the occupancy of PDE6γ, cGMP remained bound in the GAF A domains. It has been well reported that cGMP dissociation from the GAF A domains decreases the binding affinity for PDE6γ; however, in each of the structures reported here, there is strong cryo-EM density for cGMP in the GAF A domain of both PDE6 catalytic subunits. In the cGMP-bound and IBMX-bound complexes, analysis of the PDE6 conformational ensemble with 3DVA suggests that there can be asymmetric association of PDE6γ to the two catalytic domains, with a preference for the PDE6α catalytic subunit. The functional relevance of this asymmetry will be the focus of future studies; however, some form of asymmetric behavior occurs in the engagement of PDE6γ with the PDE6α and PDE6β subunits (46, 47). Despite this, several high-resolution cryo-EM structures (PDBs: 6MZB, 7JSN, four from this manuscript) have found that the nature of the binding interactions between PDE6γ appear to be similar for both catalytic subunits. Here, using 3DVA, we have been able to observe structural pictures of PDE6γ that the basis of the asymmetry may involve the association between the PDE6α and PDE6β subunits.

Taken together, these data indicate that binding of sildenafil class inhibitors decreases the affinity of the PDE6γ C terminus for the PDE6 catalytic core and allows the Gα_T_ subunit to bind PDE6γ, thus stabilizing a 2:1 Gα_T_–PDE6 complex. The stoichiometry of an active Gα_T_–PDE6 complex has been contested; however, our biochemical and structural evidence indicates that the PDE6 heterotetramer is capable of binding to two Gα_T_ simultaneously. Several studies have suggested that a 1:1 Gα_T_–PDE6 complex can achieve maximal activity, and in the presence of IBMX, we observed the formation of both 1:1 and 2:1 retinal Gα_T_·GTPγS–PDE6 complexes. Future studies will be focused on determining the structure of the Gα_T_–PDE6 complex in the presence of a lipid bilayer to capture the physiological state of this signaling complex more accurately. Importantly, the presence of a lipid bilayer provides a substantial boost to Gα_T_ stimulated PDE6 activation, which can be attributed to an increase in the apparent affinity of the interaction, likely the result of the membrane providing a platform that effectively increases the concentration of the reactants. We have probed the mechanism responsible for the membrane-induced enhancement of PDE6 activation by using lipid nanodiscs as membrane mimetics. We found that in the absence of the lipid modification on Gα_T_, the membrane-stimulated boost in PDE6 activity is abolished, suggesting that the N-terminal myristylation is critical for maintaining high levels of PDE6 activity. Interestingly, we found that the lipid nanodiscs were unable to mimic the same high-affinity boost in PDE6 activity that is achieved by the native ROS membranes. Previous studies have suggested that the retinal proteins occupy distinct regions of the ROS disc membranes, and that the lipid composition of the disc membrane is highly variable between regions (48, 49). Initial attempts were made to boost PDE6 activity by modifying the lipid composition of our nanodiscs to more closely match the native environment, but we found that this had limited effect on PDE6 activity. Through these initial studies, it became clear that it was not fruitful to explore the vast parameter space that would have to be carefully characterized to identify the role of lipid composition in our reconstituted system. In addition, the potential role for signaling lipids such as phosphoinositides, sphingolipids, and cholesterol in promoting physiological PDE6 activity has not been well-characterized. This provided the motivation to pursue the Ni-NTA (DGS) nanodiscs as a potential mimetic system for an ideal, that is, ultra-high affinity, interaction between the Gα_T_–PDE6 complex and the lipid bilayer. We found that we could fully rescue membrane stimulated PDE6 activity by functionalizing the lipid bilayer using DGS Ni(NTA) lipids, driving a high affinity interaction between Gα_T_∗ and the membrane through the N-terminal 6x-His tag. A recent review from Gulati and Palczewski suggested that PDE6 may play a critical role in defining the distance between adjacent ROS membrane leaflets by spanning across adjacent membrane bilayers (37). Based on our studies, we have determined that a critical component of Gα_T_ stimulated PDE6 activation is the ability of Gα_T_ to cause a striking repositioning of the PDE6γ C terminus at a significant distance from the active sites of PDE6αβ. In addition, we found that tightly anchoring Gα_T_ to the membrane provides a significant boost in the formation of a stable Gα_T_–PDE6 complex, resulting in the marked increase in transducin stimulated PDE6 catalytic activity. Taken together, we believe these results support the hypothesis introduced by Gulati and Palczewski and that the membrane-stimulated boost in PDE6 activity arises because of an increased ability of the anchored Gα_T_ subunits to maintain the repositioning of the PDE6γ subunit (Fig. 8*B*). Future studies will be focused on structural characterization of membrane bound Gα_T_–PDE6 complexes using membrane mimetic systems such as nanodiscs, as well as high-resolution cryo-electron tomography, and these will be critical to understanding the role of the membrane in PDE6 activation and the resulting signal amplification.

## Experimental procedures

### Purification of native ROS membrane proteins

Retinal proteins (PDE6, Gα_T_, and Gβγ) were purified from dark-adapted bovine retina as described previously. Briefly, three hundred retinas (W.L. Lawson Co) were light-exposed, mechanically disrupted, and subjected to sucrose gradient centrifugation to isolate the ROS membranes. The purified ROS were washed three times with 50 ml isotonic buffer (10 mM Hepes pH 7.5, 100 mM NaCl, 5 mM MgCl_2_, 0.1 mM EDTA, and 1 mM DTT) and PDE6 was isolated with three washes (50 ml) of hypotonic buffer (10 mM Hepes pH 7.5, 0.1 mM EDTA, 1 mM DTT). PDE6 was purified from the hypotonic wash by anion exchange chromatography using a 1 ml HiTrap Q HP column (Cytiva Life Sciences) and eluted over a gradient of Buffer A (20 mM Tris pH 8, 5 mM MgCl_2_, 1 mM DTT) and buffer B (buffer A with 1 M NaCl). The purified PDE6 was then injected onto a Superdex 200 Increase 10/300 Gl (Cytiva Life Sciences) equilibrated with PDE6 storage buffer (20 mM Tris pH 8, 100 mM NaCl, 10% glycerol, and 1 mM DTT) for purification by size-exclusion chromatography. PDE6 was concentrated to 15 μM with a 100 kDa MW cut-off centrifugal filter unit (Amicon, Millipore Sigma), flash froze in liquid nitrogen, and stored at −80 °C. Protein concentrations were determined using the extinction coefficient calculated with the ExPASy ProtParam tool. The retinal Gα_T_ and Gβγ subunits were isolated by further washing the ROS with 100 ml of GTP buffer (10 mM Hepes pH 7.5, 0.1 mM EDTA, 100 μM GTP, 1 mM DTT). The GTP wash was loaded onto two 5 ml HiTrap Blue HP (Cytiva Life Sciences) to separate the Gα_T_ and Gβγ subunits, which were then further purified separately by anion exchange chromatography using a 5 ml HiTrap Q HP (Cytiva Life Sciences) and buffer A (20 mM Hepes pH 7.5, 5 mM MgCl_2_, 1 mM DTT, 10% glycerol) and buffer B (buffer A with 1 M NaCl) to form the gradient. Both transducin subunits were concentrated to 20 μM, flash froze in liquid nitrogen and stored at −80 °C.

### Expression and purification of recombinant Gα_T_∗

Recombinant Gα_T_∗ was expressed and purified as previously described. Briefly, *E. coli* BL21 (DE3) competent cells (New England Biolabs) were transformed with the plasmid encoding the pHis6 Chi8HN QLRC SFD. The *E. coli* cells were grown in six liters of LB with 100 μg/ml ampicillin at 37 °C to an *A*_600_ of 0.6 to 0.8, and protein expression was induced at 19 °C with 30 μM IPTG for 16 to 18 h. The cells were resuspended in lysis buffer (50 mM Tris pH 8, 500 mM NaCl, 5 mM MgCl_2_, 0.1 mM PMSF, 5 mM β-mercaptoethanol (BME), 10% glycerol, 50 μM GTP) and disrupted by sonication. The lysate was clarified by centrifugation at 185,000*g* for 45 min. The supernatant was then loaded onto a 5 ml HisTrap HP column (Cytiva Life Sciences) equilibrated with equilibration buffer (50 mM Tris pH 8, 500 mM NaCl, 20 mM imidazole, 5 mM BME), washed with 100 ml of equilibration buffer, and then washed again with 100 ml of wash buffer (50 mM Tris pH 8, 20 mM imidazole, 5 mM BME). The protein was eluted with 50 ml of the wash buffer containing 250 mM imidazole. The eluent was loaded onto a 5 ml HiTrap Q HP column (Cytiva Life Sciences) and eluted with a gradient of buffer A (20 mM Hepes pH 7.5, 5 mM MgCl_2_, 1 mM DTT, 10% glycerol) and buffer B (buffer A with 1 M NaCl). The Gα_T_∗ was then concentrated to 20 μM, flash froze in liquid nitrogen and stored at −80 °C.

### Nanodisc preparation

Membrane scaffolding proteins were expressed and purified as previously described (50, 51, 52). Lipids were purchased from Avanti as chloroform dissolved lipids and were prepared by evaporating the chloroform, desiccating in a vacuum desiccator overnight, and resuspending in a buffer containing 20 mM Tris pH 7.5, 100 mM NaCl, 0.5 mM EDTA, and 100 mM sodium cholate. Purified tobacco etch virus protease-cleaved membrane scaffolding protein and detergent solubilized lipids were then mixed at the ratios described previously and incubated on ice for 2 h. BioBeads were then added to remove detergent and the prepared nanodiscs were purified using a Superdex 200 Increase 10/300 Gl (Cytiva Life Sciences). The purified nanodiscs were evaluated for homogeneity *via* inspection of the chromatogram and with negative stain EM. For negative stain EM, the nanodisc samples were diluted to 0.01 mg/ml and 10 μl was deposited onto a freshly glow discharged carbon/formvar coated copper EM grid. The excess sample was blotted, washed with 3 × 10 μl of milliQ water, and stained with 3 × 10 μl 2% uranyl acetate. Images were collected on a Morgagni (100 kV) at a magnification of 89,000× and samples were visually inspected for homogeneity.

### PDE activity assay

Hydrolysis of cGMP by PDE6 was measured and processed as previously described (53). Briefly, 50 nM PDE6 and varying amounts of GTPγS-loaded retinal Gα_T_ or the recombinant Gα_T_∗ was preincubated in a buffer containing 10 mM Tris pH 8, 2 mM MgCl_2_, and 100 mM NaCl. Membrane stimulated activity was measured using UROS membranes containing 5 μM rhodopsin or 400 nM of reconstituted nanodiscs. The pH change resulting from cGMP hydrolysis was measured as a change in mV in real time using a micro pH electrode with a SevenDirect pH meter (Mettler Toledo). The buffering capacity of the solution was measured by addition of NaOH, and the cGMP hydrolysis rate (mol/sec) was determined from the ratio of the initial rate of pH change (mV/sec) and the buffering capacity of the assay mixture (mV/mol). Enzyme activity was normalized using the activity of trypsinized PDE6, which has been previously shown to represent maximally active PDE6.

### Cryo-EM sample preparation and data collection

The apo-PDE6 sample was prepared by incubating 6 μM PDE6 with 12 μM Gα_T_∗ (1.8 mg/ml Gα_T_∗–PDE6 complex) for 10 min on ice. Cryo-EM samples were frozen using a Vitrobot Mark IV (Thermo Fisher Scientific) maintained at 4 °C and 100% humidity. A 4.2 μl solution was applied to glow-discharged gold holey carbon grids (Quantifoil, Au R1.2/1.3, 200 mesh) and the excess sample was blotted away for 2 s, then plunge-frozen into liquid ethane cooled with liquid nitrogen. Cryo-EM images were collected on a Talos Arctica (Thermo Fisher Scientific) with a Gatan GIF Quantum LS Imaging energy filter (20 kV slit), operated at 200 kV at a nominal magnification of 79,000× with a corresponding super-resolution pixel size of 0.517 Å and a 70 μm objective lens. Micrographs were recorded using a Gatan K3 direct electron detector with a dose rate of 27.5 electrons/pixel/s for a total dose of 50 electrons/Å2 and defocus values ranging from −1.2 μm to −2.4 μm. Images were collected using EPU.

The PDE6-udenafil complex was prepared by incubating 0.7 mg/ml PDE6 with 20 μM udenafil for 5 min. Cryo-EM samples were frozen using a Vitrobot Mark IV (Thermo Fisher Scientific) maintained at 4 °C and 100% humidity. A 4.2 μl solution was applied to glow-discharged gold holey carbon grids (Quantifoil, Au R1.2/1.3, 200 mesh) and the excess sample was blotted away for 2 s, then plunge-frozen into liquid ethane cooled with liquid nitrogen. Cryo-EM images were collected on a Talos Arctica (Thermo Fisher Scientific) with a Gatan GIF Quantum LS Imaging energy filter (20 kV slit), operated at 200 kV at a nominal magnification of 63,000× with a corresponding size of 1.31 Å and a 100 μm objective lens. Micrographs were recorded using a Gatan K3 direct electron detector with a dose rate of 24.23 electrons/pixel/s for a total dose of 50 electrons/Å2 and defocus values ranging from −1.4 μm to −2.0 μm. Images were collected using 3 × 3 multishot acquisition in SerialEM.

The PDE6–cGMP complex was prepared by incubating 3 μM PDE6 with 10 μM Gα_T_∗ for 5 min, followed by the addition of 5 mM cGMP and immediate plunge freezing (within 10 s) of the grids. Cryo-EM samples were frozen using a Vitrobot Mark IV (Thermo Fisher Scientific) maintained at 4 °C and 100% humidity. A 4.2 μl solution was applied to glow-discharged gold holey carbon grids (Quantifoil, Au R1.2/1.3, 200 mesh) and the excess sample was blotted away for 2 s, then plunge-frozen into liquid ethane cooled with liquid nitrogen. Cryo-EM images were collected on a Talos Arctica (Thermo Fisher Scientific) with a Gatan GIF Quantum LS Imaging energy filter (20 kV slit), operated at 200 kV at a nominal magnification of 63,000× with a corresponding pixel size of 1.31 Å and a 100 μm objective lens. Micrographs were recorded using a Gatan K3 direct electron detector with a dose rate of 26.03 electrons/pixel/s for a total dose of 50 electrons/Å2 and defocus values ranging from −1.4 μm to −2.0 μm. Images were collected using 3 × 3 multishot acquisition in SerialEM.

The PDE6–IBMX complex was prepared by incubating 3 μM PDE6 with 20 μM retinal Gα_T_·GTPγS for 5 min, followed by the addition of 20 μM IBMX. Cryo-EM samples were frozen using a Vitrobot Mark IV (Thermo Fisher Scientific) maintained at 4 °C and 100% humidity. A 4.2 μl solution was applied to glow-discharged gold holey carbon grids (Quantifoil, Au R1.2/1.3, 200 mesh) and the excess sample was blotted away for 2 s, then plunge-frozen into liquid ethane cooled with liquid nitrogen. Cryo-EM images were collected on a Talos Arctica (Thermo Fisher Scientific) with a Gatan GIF Quantum LS Imaging energy filter (20 kV slit), operated at 200 kV at a nominal magnification of 63,000× with a corresponding pixel size of 1.34 Å and a 100 μm objective lens. Micrographs were recorded using a Gatan K3 direct electron detector with a dose rate of 27.25 electrons/pixel/s for a total dose of 50 electrons/Å2 and defocus values ranging from −1.0 μm to −2.6 μm. Images were collected using 3 × 3 multishot acquisition in SerialEM.

### Cryo-EM data processing

All cryo-EM data processing was carried out using cryoSPARC version 4.2.1 (Structura Biotechnology) (54). Workflows are included in the Supplementary (Figs. S10–S13). Briefly, Patch Motion Correction and Patch contrast transfer function (CTF) estimation were used to prepare the micrographs. Micrographs were curated to include CTF fits better than 5 to 6 Å and a relative ice thickness of greater than one and less than 1.05 for downstream processing. Blob picking with a maximum radius of 160 Å was used to generate templates, which were then used for template picking. 2D classification was used to select particles that displayed clear secondary structure features and these particles were used for downstream processing. Selected particles were used in iterative rounds of *ab initio* reconstruction and heterogeneous refinement to investigate heterogeneity in the samples. Homogeneous refinement and nonuniform refinement were then used to generate a consensus map (55). Defocus refinement, Global CTF, and local CTF refinement were carried out as implemented in cryoSPARC (56). The consensus volume was then used as input for 3DVA, where the results were filtered to 6 Å (23). Cryo-EM maps were sharpened using sharpening tools in cryoSPARC.

### Model building, refinement, and validation

All initial atomic models were generated with ModelAngelo (version 1.0.1) using the sharpened cryo-EM maps and the protein sequences for bovine PDE6 as defined in UniProt (PDE6A: P11541, PDE6B: P23439, PDE6G: P04792) as input. The ModelAngelo outputs were then inspected and refined manually in WinCoot (Coot version 0.9.4.1, https://bernhardcl.github.io/coot/wincoot-download.html) and then further refined using Phenix Real Space Refinement (Phenix Version 1.20.1). To generate a model of the retinal Gα_T_·GTPγS–PDE6 complexes, the crystal structure of retinal Gα_T_ bound to GTPγS (PDB: 1TND) was aligned to and substituted for the Gα_T_∗ subunits from the atomic model of the chimeric Gα_T_∗–PDE6 complex from Gao *et al.* (PDB: 7JSN) (27).

## Data availability

All structural data has been deposited to the PDB and EMDB with accession numbers described in Table 1. The anisotropic cryo-EM maps that are not associated with atomic models have been deposited to the EMDB as follows: PDE6 bound to one retinal Gα_T_·GTPγS: EMD-42235, PDE6 bound to two retinal Gα_T_·GTPγS: EMD-42237, and PDE6 bound to two chimera Gα_T_∗ without a stabilizing antibody: EMD-42238. All other data is contained within the manuscript and associated Supporting information.

## Supporting information

This article contains supporting information.

## Conflict of interest

The authors declare that they have no conflict of interest with the contents of this article.
