# Supplementary material for: Probing the mechanism by which the retinal G protein transducin activates its biological effector PDE6
Source: J Biol Chem. 2023 Dec 28;300(2):105608. doi: 10.1016/j.jbc.2023.105608 (PMC10838916; doi:10.1016/j.jbc.2023.105608)
Supplement: Supporting Figures S1–S13 [file mmc1.docx]

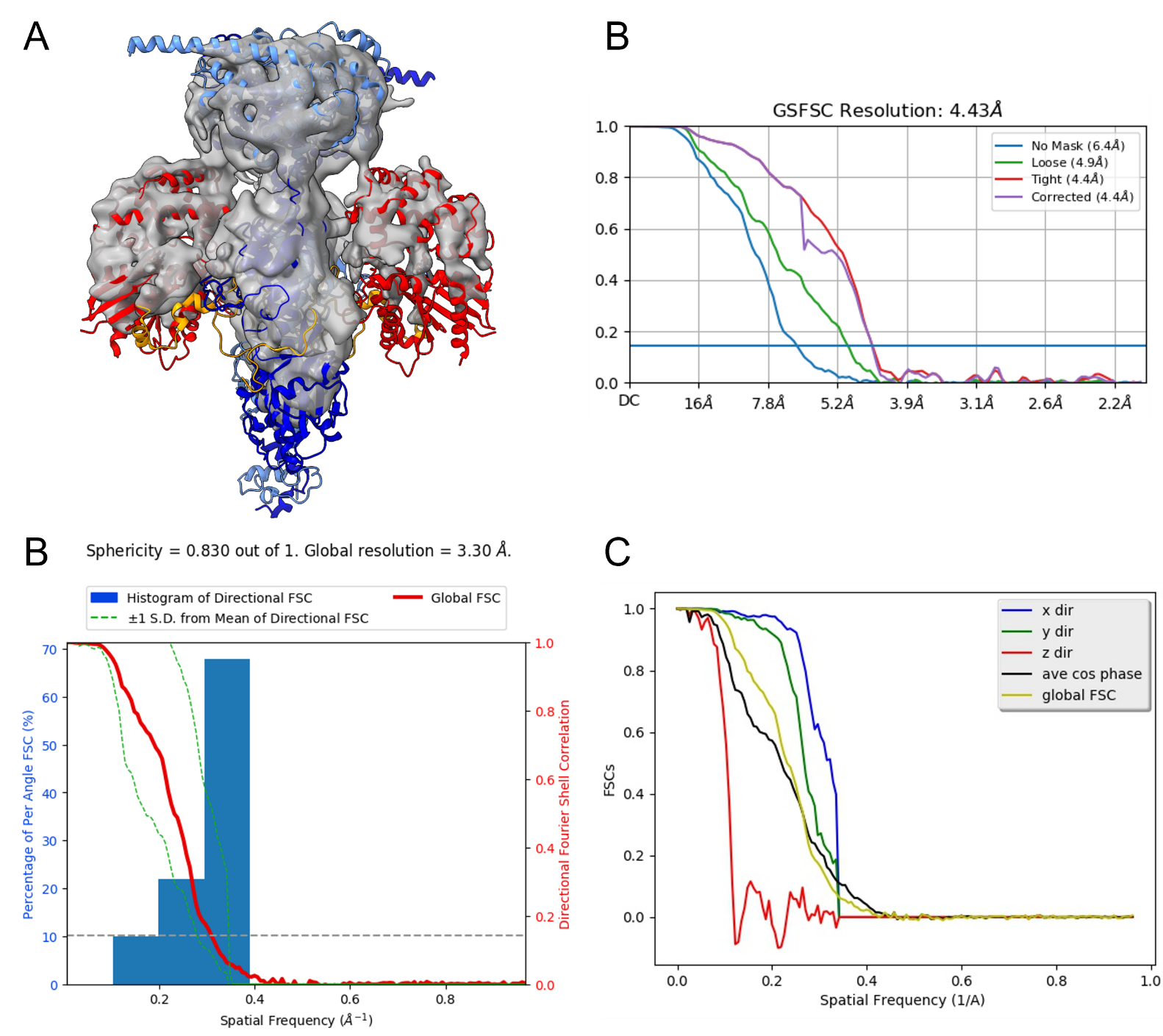


Figure S1: A) Low-resolution cryo-EM map (grey) of the Gα_T_^*^–PDE6 complex in the absence of the bivalent 1D4 antibody. Strong sample orientation bias prevents high-resolution characterization, but the stoichiometry of the complex is clear. The atomic model from Gao et al. (PDB: 7JSN) is docked into the low resolution map. PDE6α is colored dark blue, PDE6β is colored dark blue, PDE6γ is colored orange, and Gα_T_^*^ is colored red. B) Histogram and C) directional FSC plot for the 2:1 Gα_T_^*^–PDE6 complex.


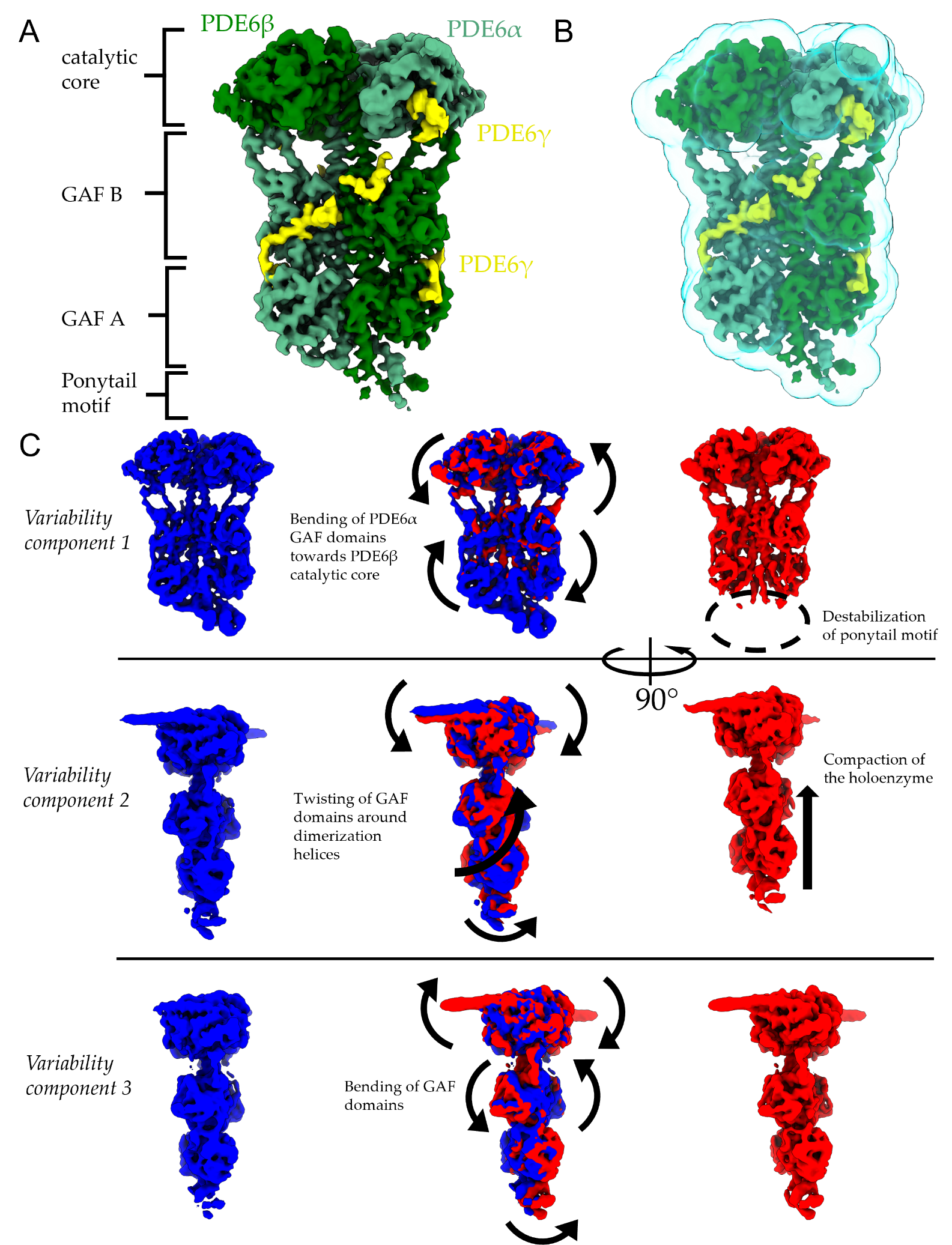


Figure S2: A) Consensus 3.1 Å cryo-EM map of apo-PDE6 shown from a front-on view of one lateral face. The PDE6αβ subunits are colored light green and dark green, respectively, and the PDE6γ subunit is colored yellow. The PDE6 domains are labeled on the PDE6 holoenzyme. B) Mask that is used for 3DVA processing. C) 3DVA to identify conformational heterogeneity in apo-PDE6. Variability component 1 resolves bending of the PDE6α GAF domains toward the PDE6β catalytic core, which is associated with destabilization of the ponytail motif. Variability component 2 resolves lateral twisting of the GAF domains around the dimerization helices, which is accommodated by movement of the catalytic core toward the GAF domains. Variability component 3 resolves bending of the GAF domains away from the catalytic core on one lateral face, which is coordinated with movement of the GAF domains toward the catalytic core of the other latter face.


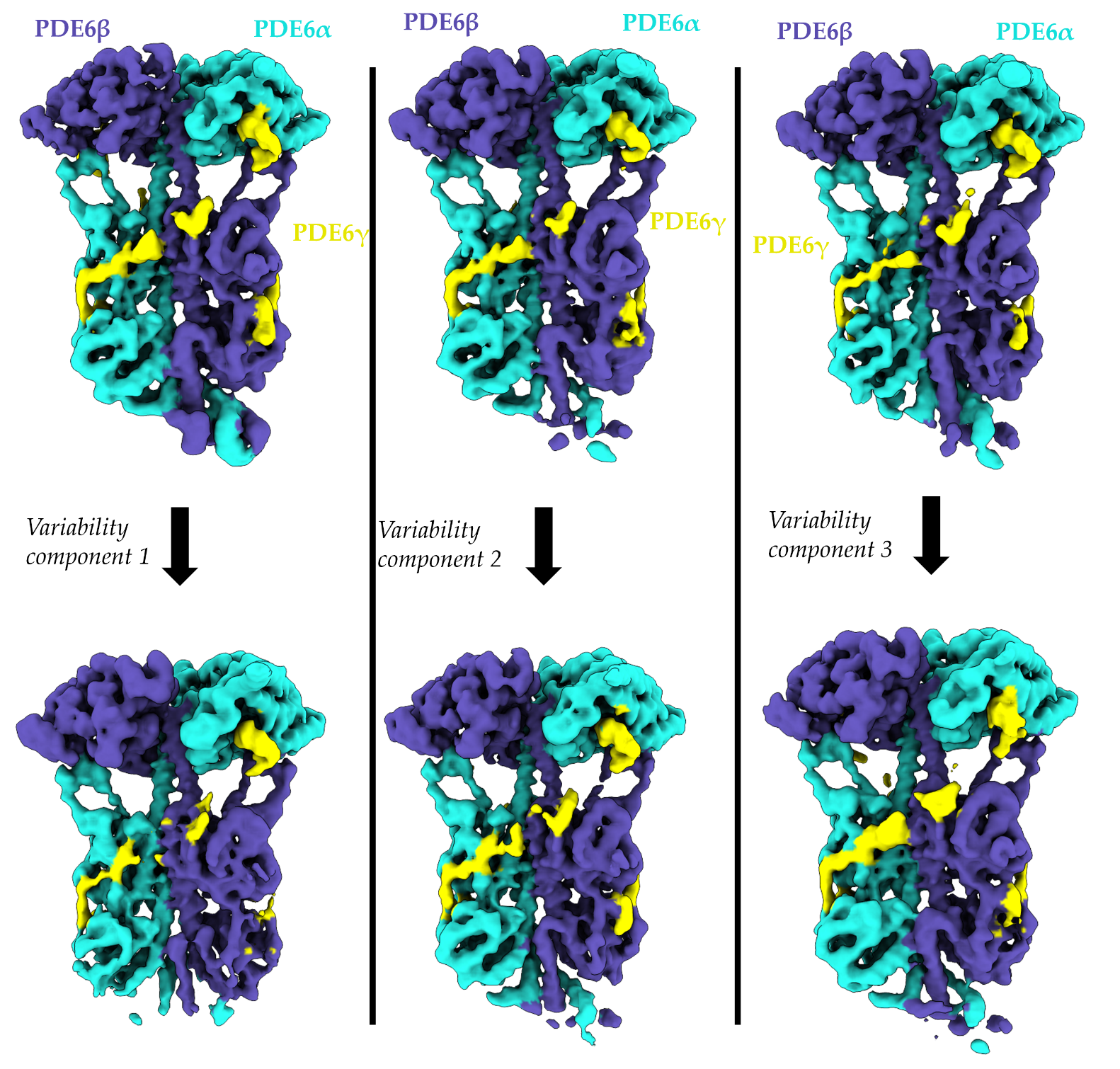


Figure S3: 3DVA analysis of the PDE6 holoenzyme shows strong association of PDE6γ with both GAF domains and the catalytic core of PDE6α/β across all variability components. Viewed across variability component 1, the ponytail region appears to be highly flexible.


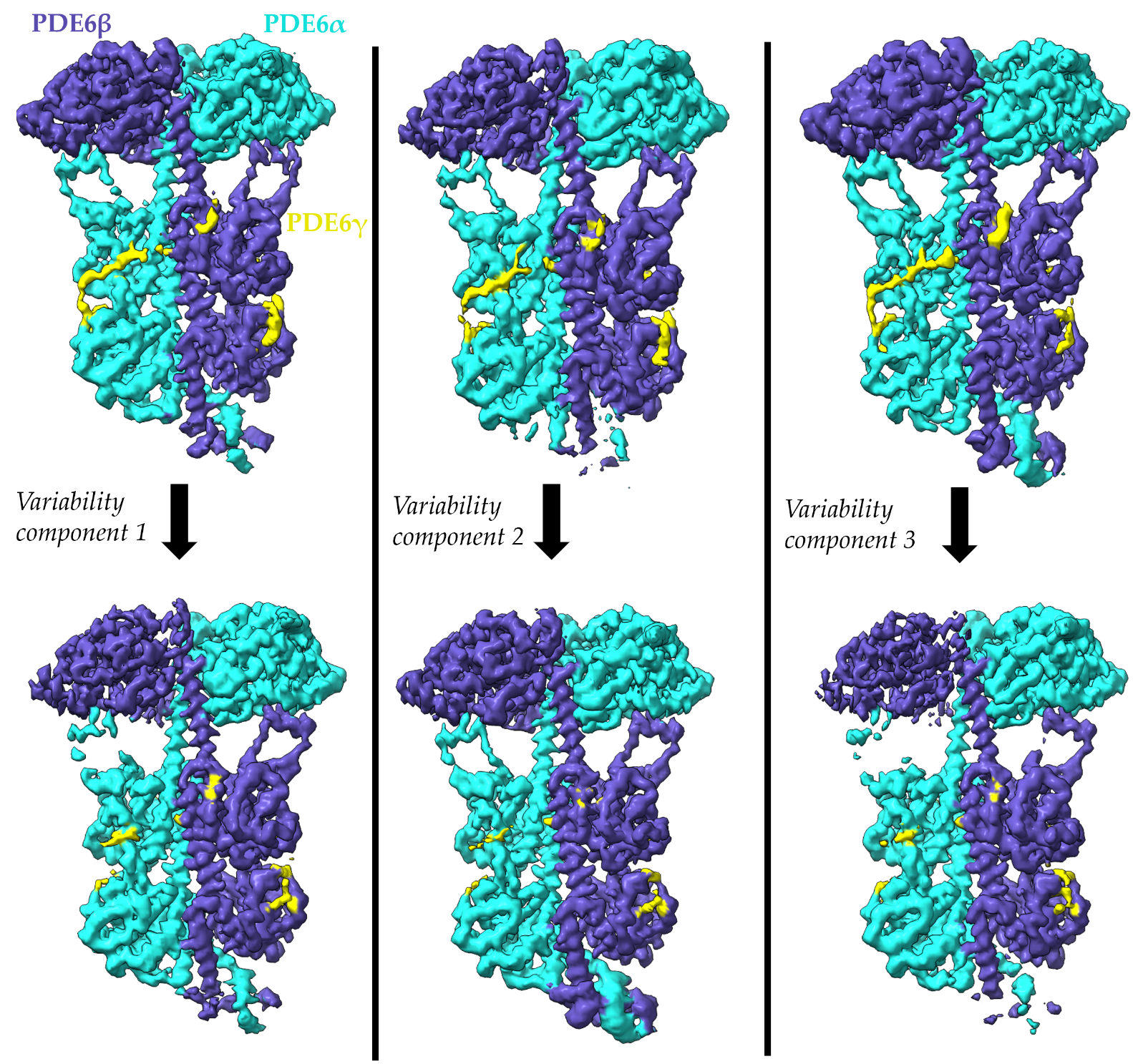


Figure S4: 3DVA analysis of the PDE6-udenafil complex shows a diminished association of PDE6γ with the GAF domains PDE6αβ across all variability components. Movement along the variability axis was visualized by comparing the top and bottom cryo-EM maps for each component. The map density for the PDE6α (cyan), PDE6β (purple), and PDE6γ (yellow) are colored within 5 Å of the atomic model.


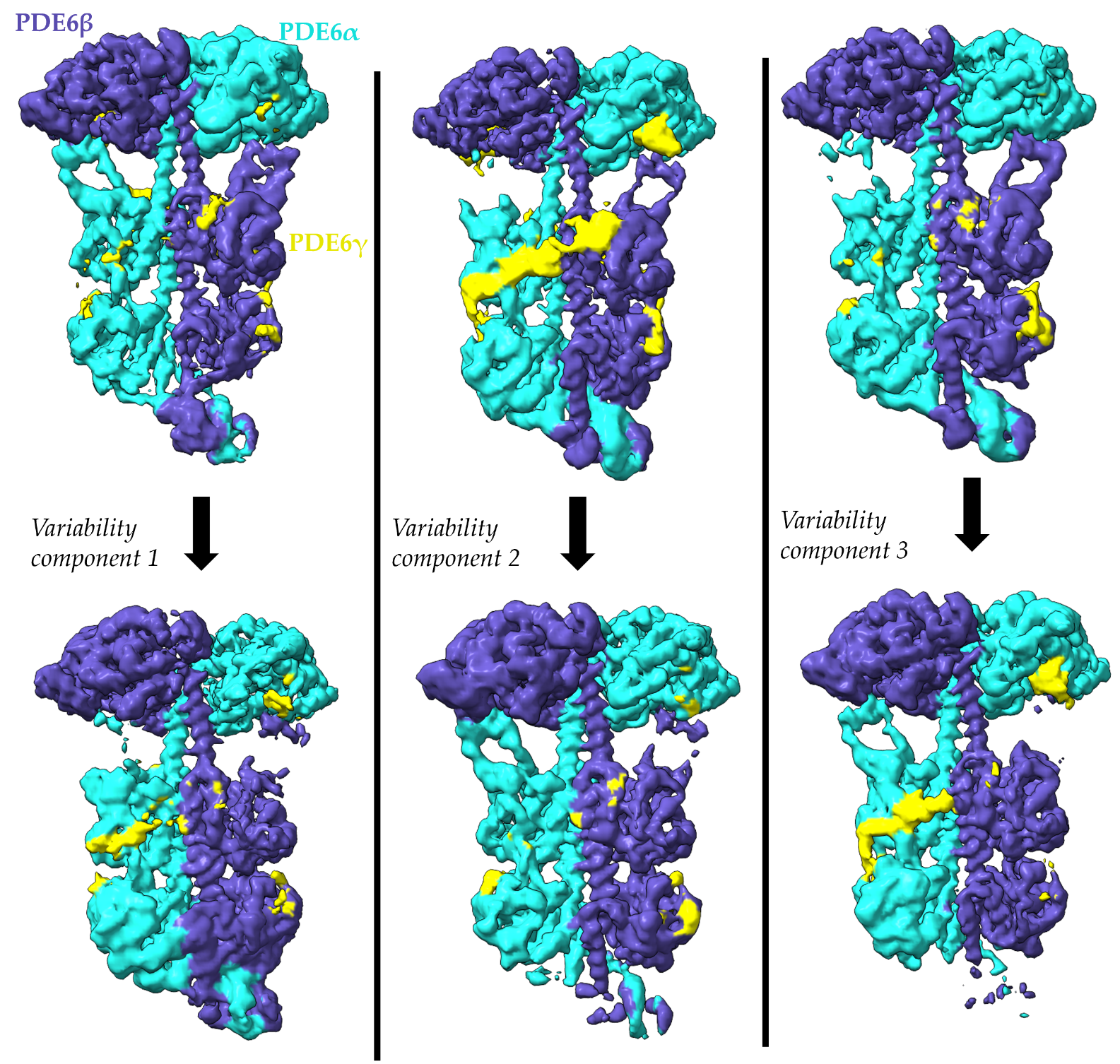


Figure S5: 3DVA analysis of the PDE6-cGMP complex shows a diminished association of PDE6γ with the GAF domains of the PDE6α and PDE6β subunits across all variability components. Movement along the variability axis was visualized by comparing the top and bottom cryo-EM maps for each component. The map density for the PDE6α (cyan), PDE6β (purple), and PDE6γ (yellow) are colored within 5 Å of the atomic model.


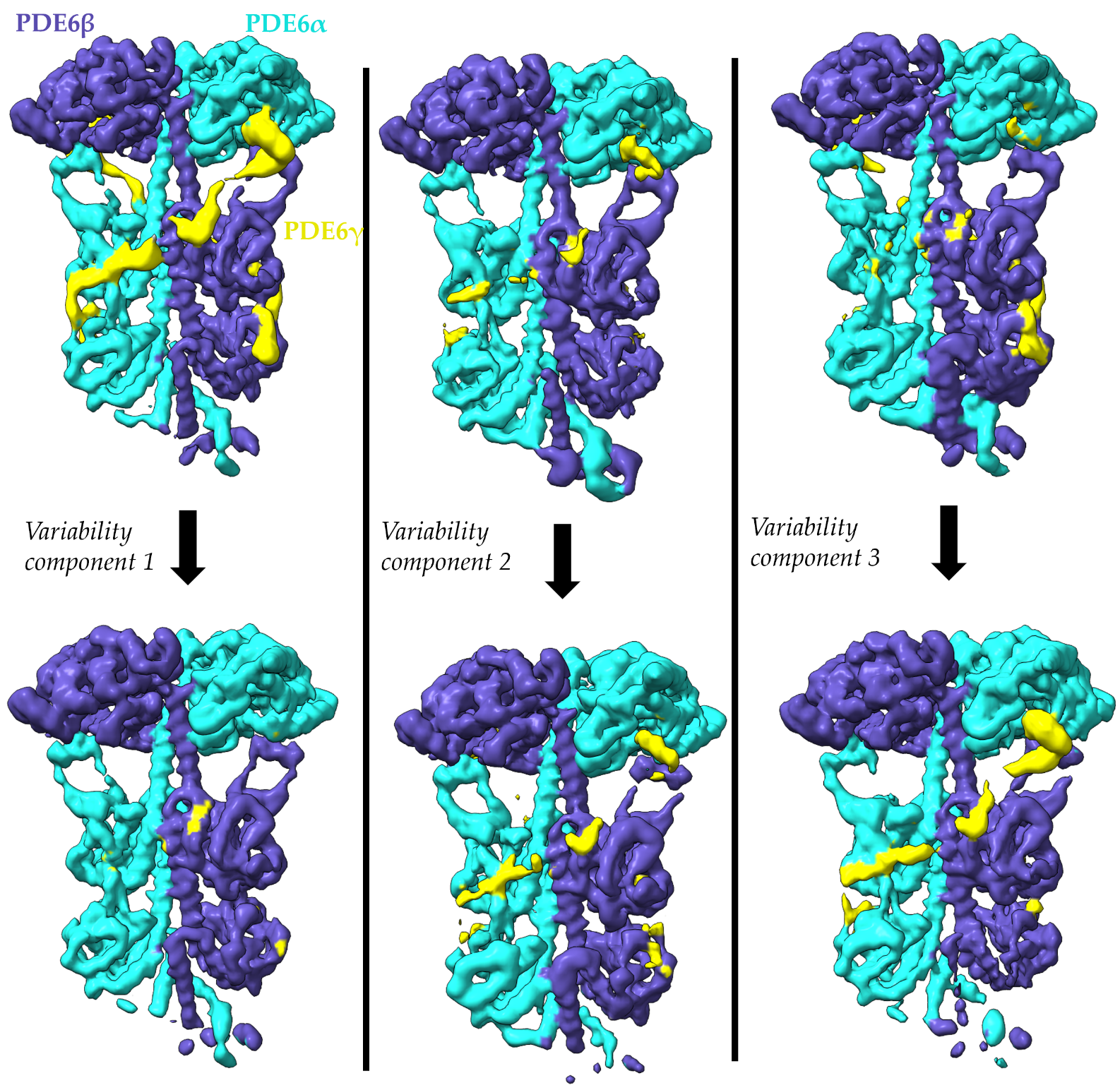


Figure S6: 3DVA analysis of the PDE6-IBMX complex shows a diminished association of PDE6γ with the GAF domains PDE6αβ across all variability components. Movement along the variability axis is visualized by comparing the top and bottom cryo-EM maps for each component. The map density for the PDE6α (cyan), PDE6β (purple), and PDE6γ (yellow) are colored within 5 Å of the atomic model.


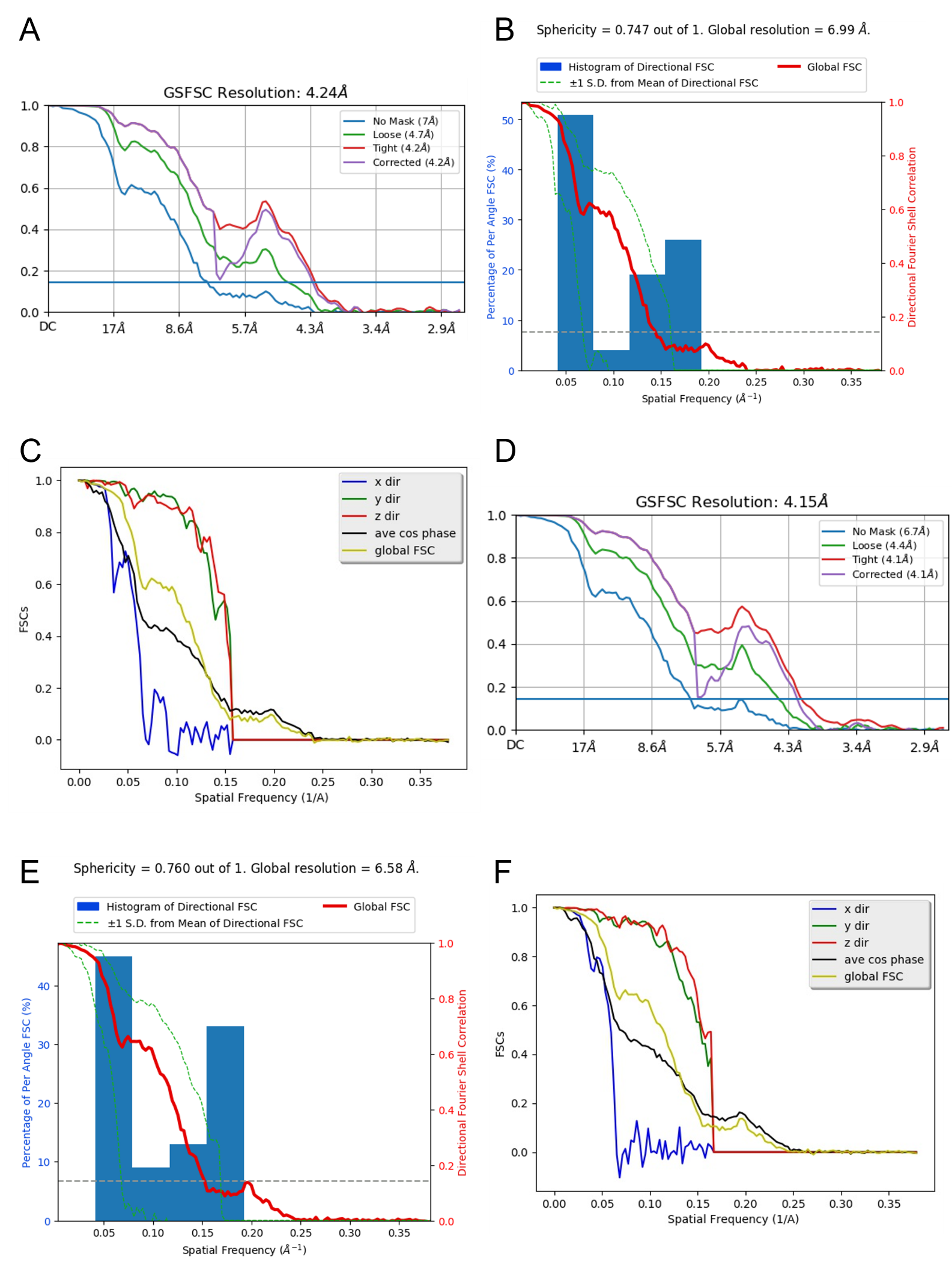


Figure S7: Gold standard FSC and 3DFSC evaluation of the 2:1 and 1:1 retinal Gα_T_·GTPγS–PDE6 complex. A) Gold standard FSC (cutoff = 0.143) from the reconstruction of the 2:1 retinal Gα_T_·GTPγS–PDE6 complex. B) Histogram and C) directional FSC plot for the 2:1 retinal Gα_T_·GTPγS–PDE6 complex. D) Gold standard FSC (cutoff = 0.143) from the reconstruction of the 1:1 retinal Gα_T_·GTPγS–PDE6 complex. E) Histogram and F) directional FSC plot for the 1:1 retinal Gα_T_·GTPγS–PDE6 complex.


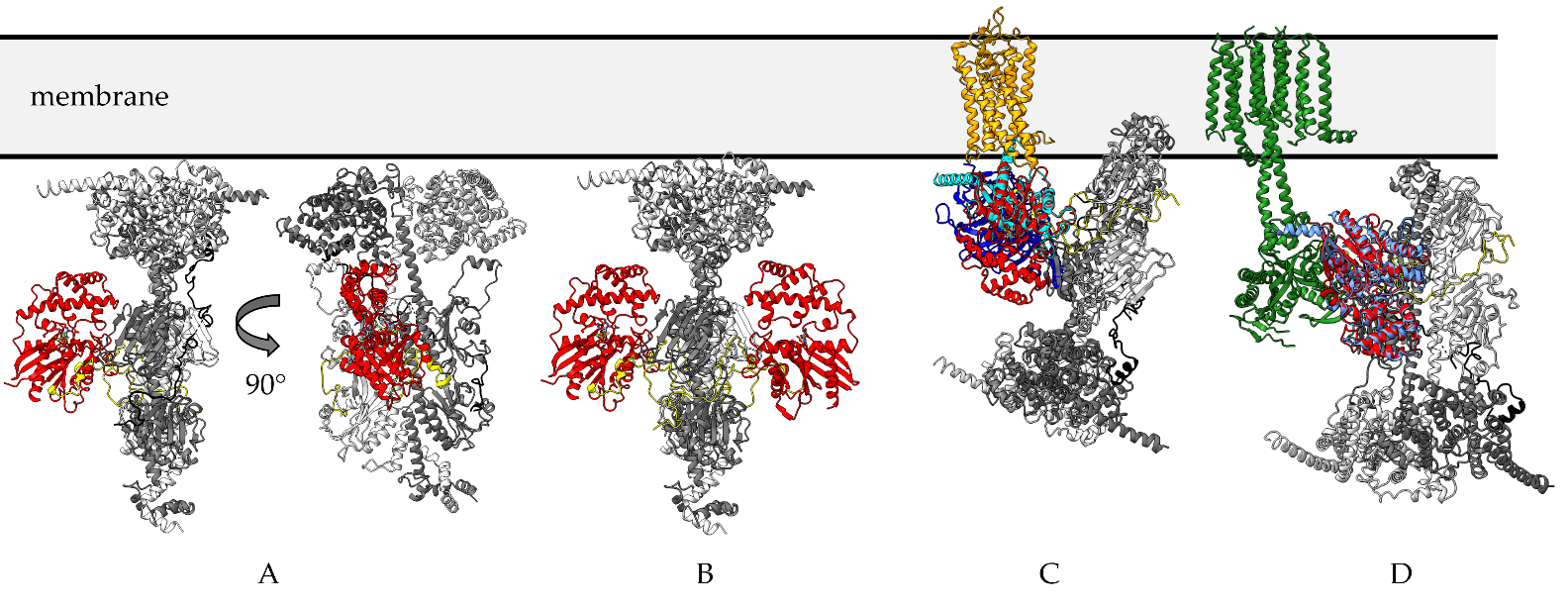


Figure S8: Comparison of the orientation of the Gα subunit in different protein complexes. A) The presumed association of the 1:1 retinal Gα_T_·GTPγS–PDE6 complex with the membrane, based on the location of lipid modifications on the C-termini of the PDE6α and PDE6β subunits. B) The presumed association of the 2:1 retinal Gα_T_·GTPγS–PDE6 complex with the membrane, based on the location of lipid modifications on the C-termini of PDE6α and PDE6β. C) Orientation of the Gα_T_ subunit in the cryo-EM structure of the rhodopsin-transducin complex (PDB: 6OY9) compared to the 1:1 retinal Gα_T_·GTPγS–PDE6 complex. The structures are aligned by the Gα_T_ subunits and membrane-bound rhodopsin is used to orient the complexes with the membrane. D) Orientation of the Gα_s_ subunit in the cryo-EM structure of adenylyl cyclase bound to Gα_s_ (PDB: 6R3Q) compared to the 1:1 retinal Gα_T_·GTPγS–PDE6 complex. The structures are aligned by the Gα subunits and membrane-bound adenylyl cyclase is used to orient the complexes with the membrane.


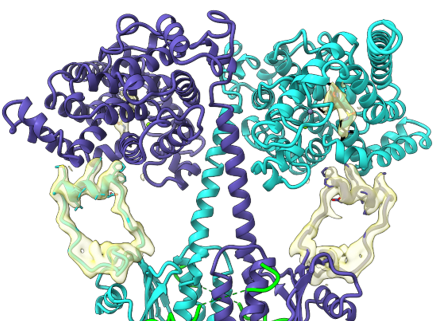


Figure S9: Cryo-EM map density for GAF B loops in the udenafil-bound PDE6 structure. The density is shown within 3 Å of the GAF B loops and udenafil.


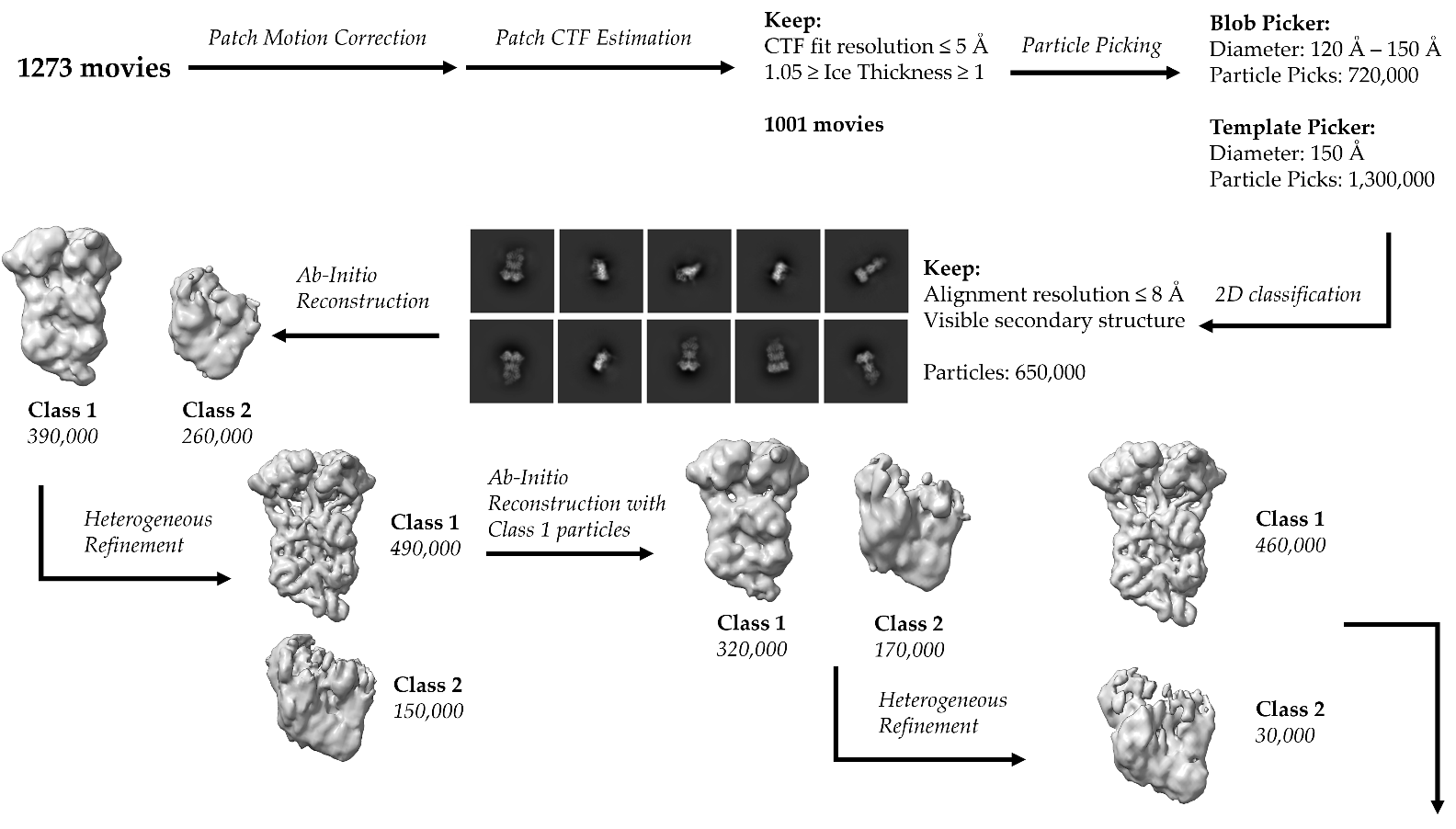

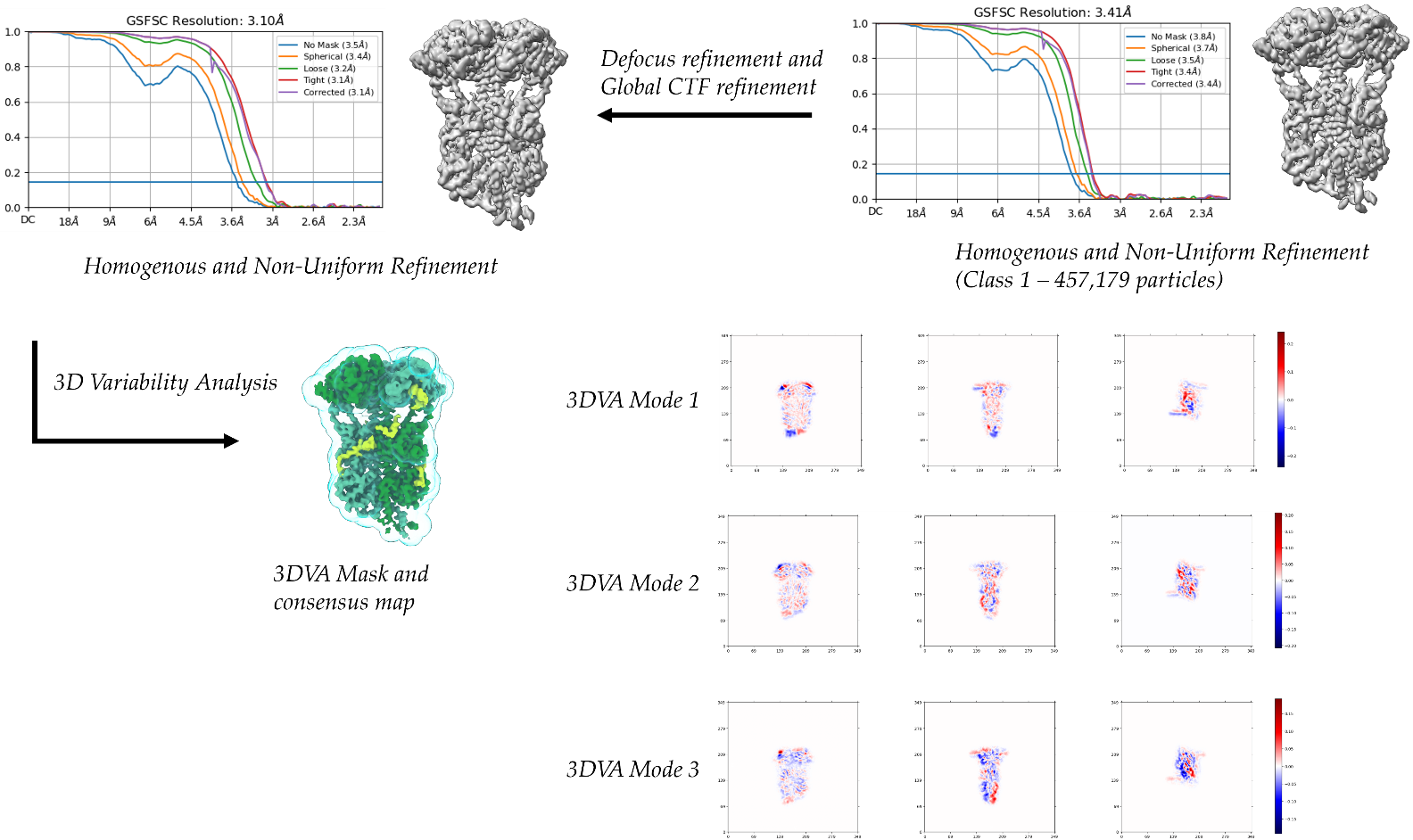


Figure S10: Cryo-EM data processing workflow for apo-PDE6.


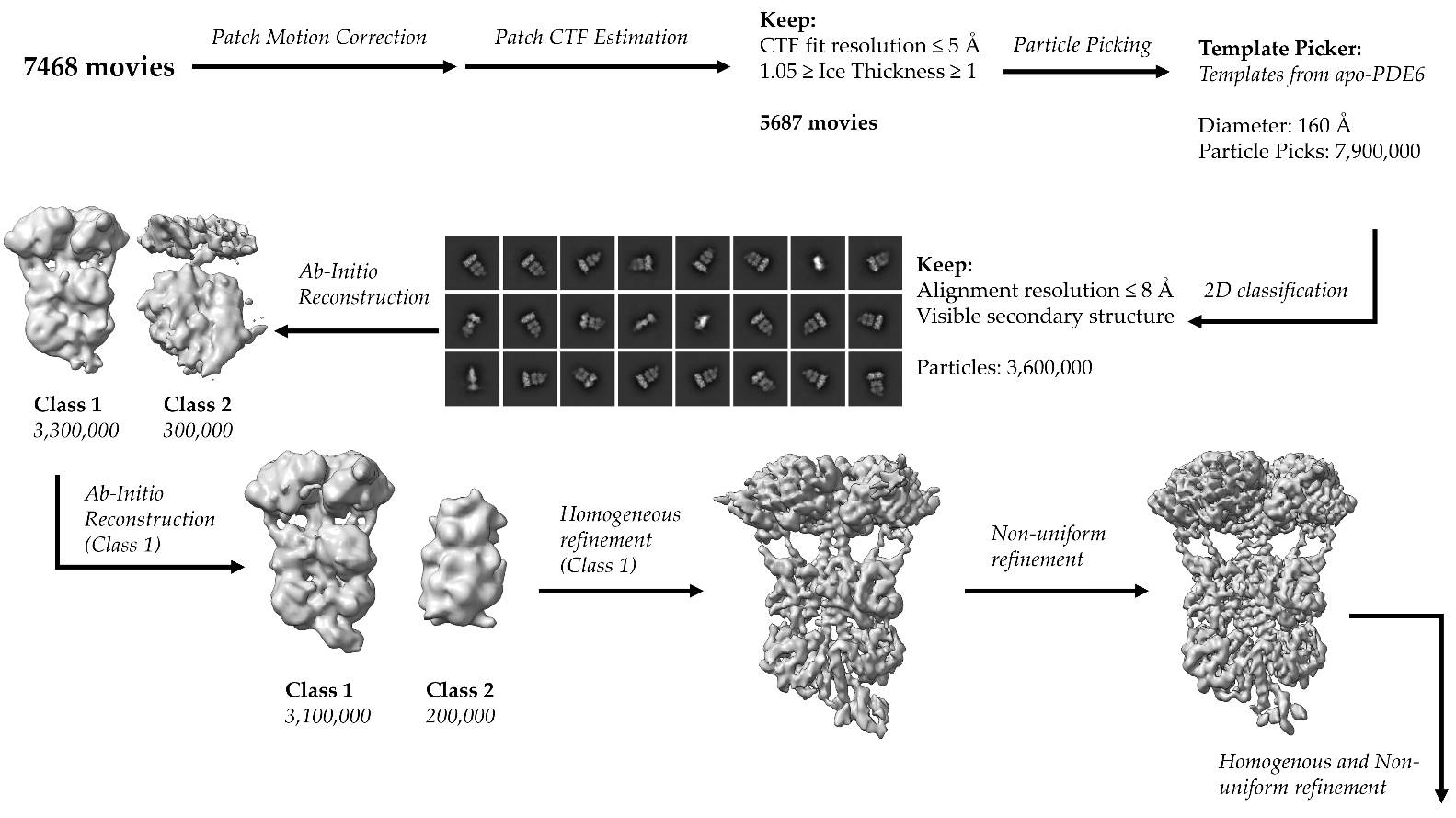

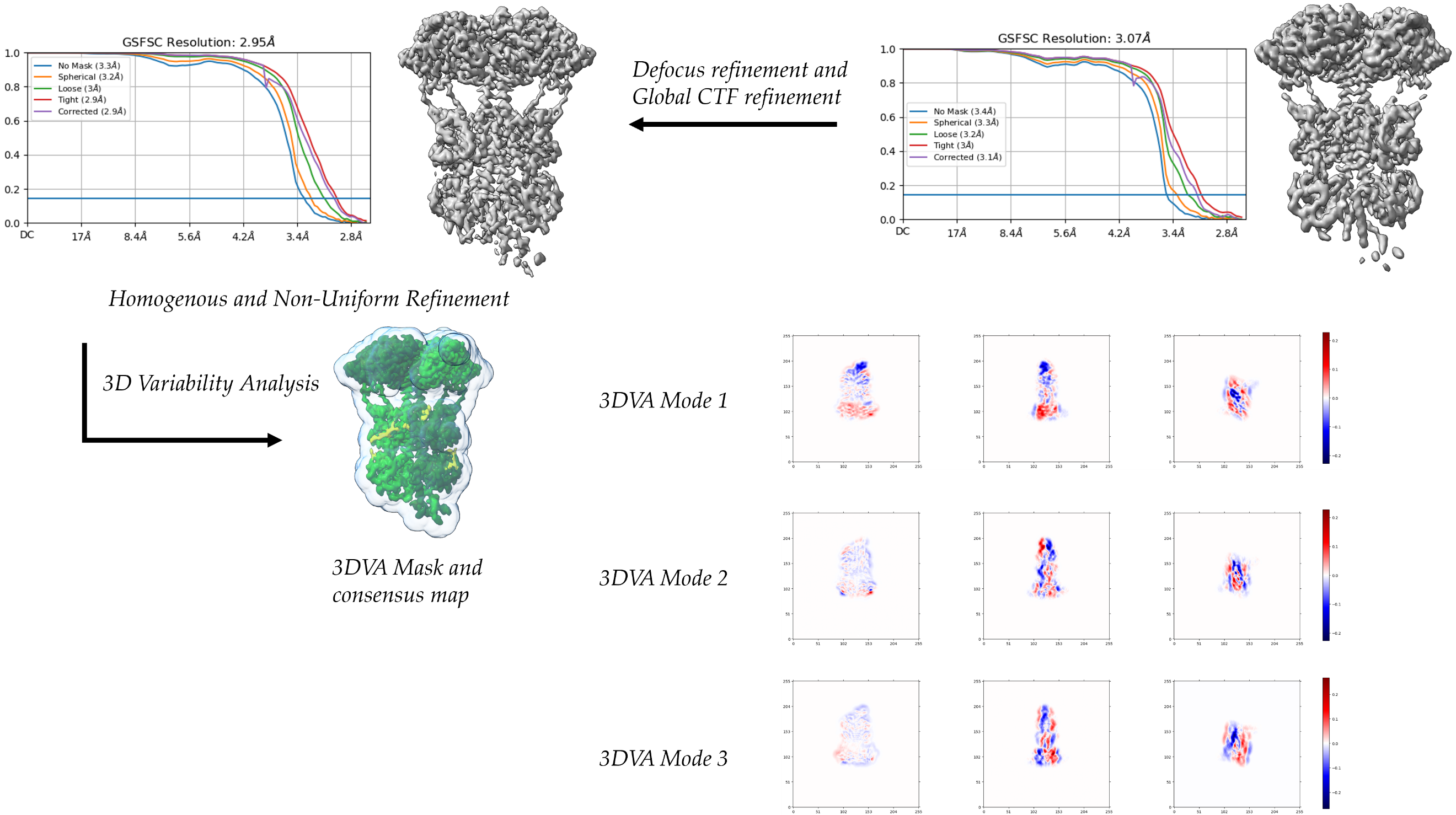


Figure S11: Cryo-EM data processing workflow for PDE6 bound to udenafil.


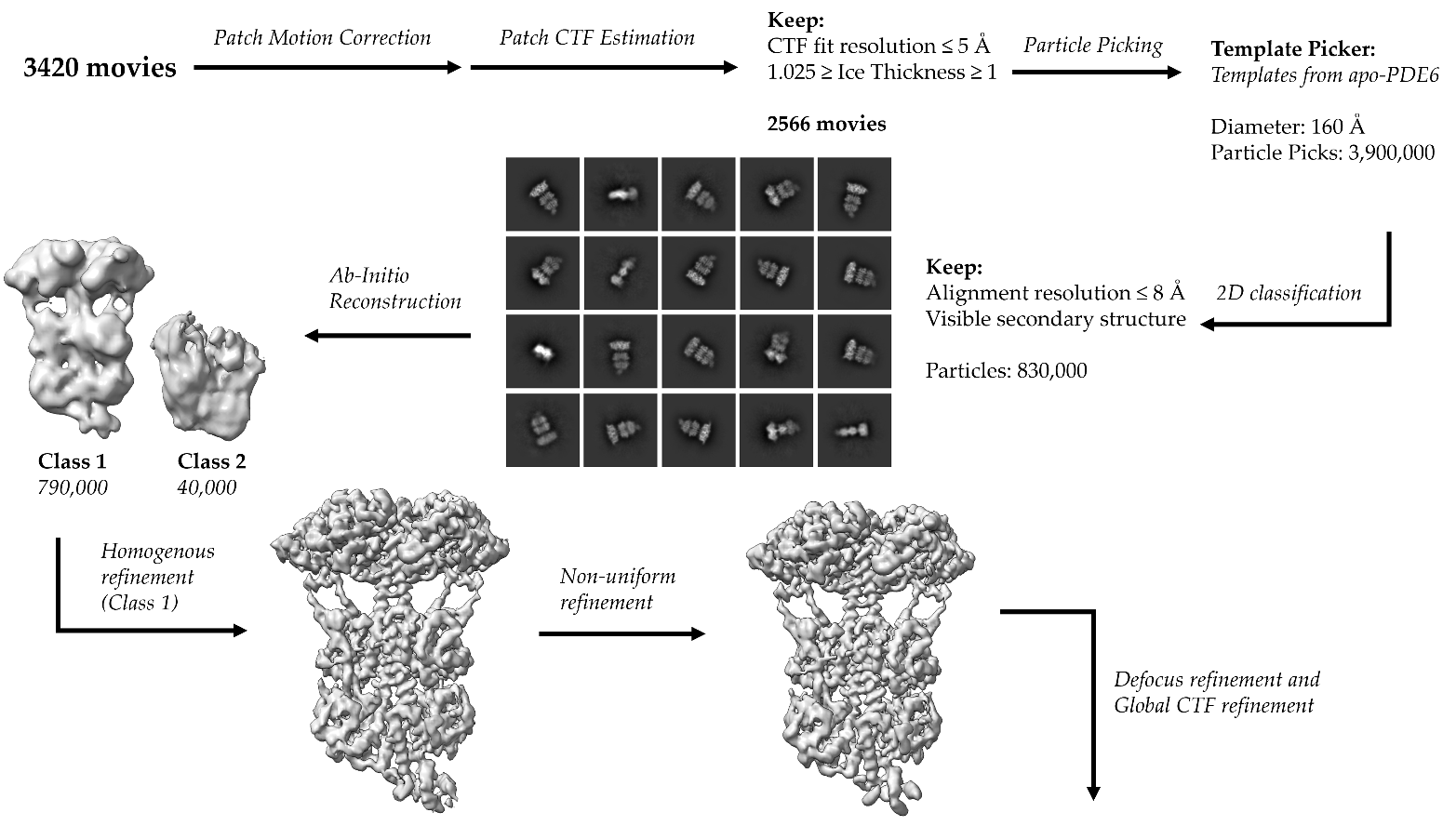

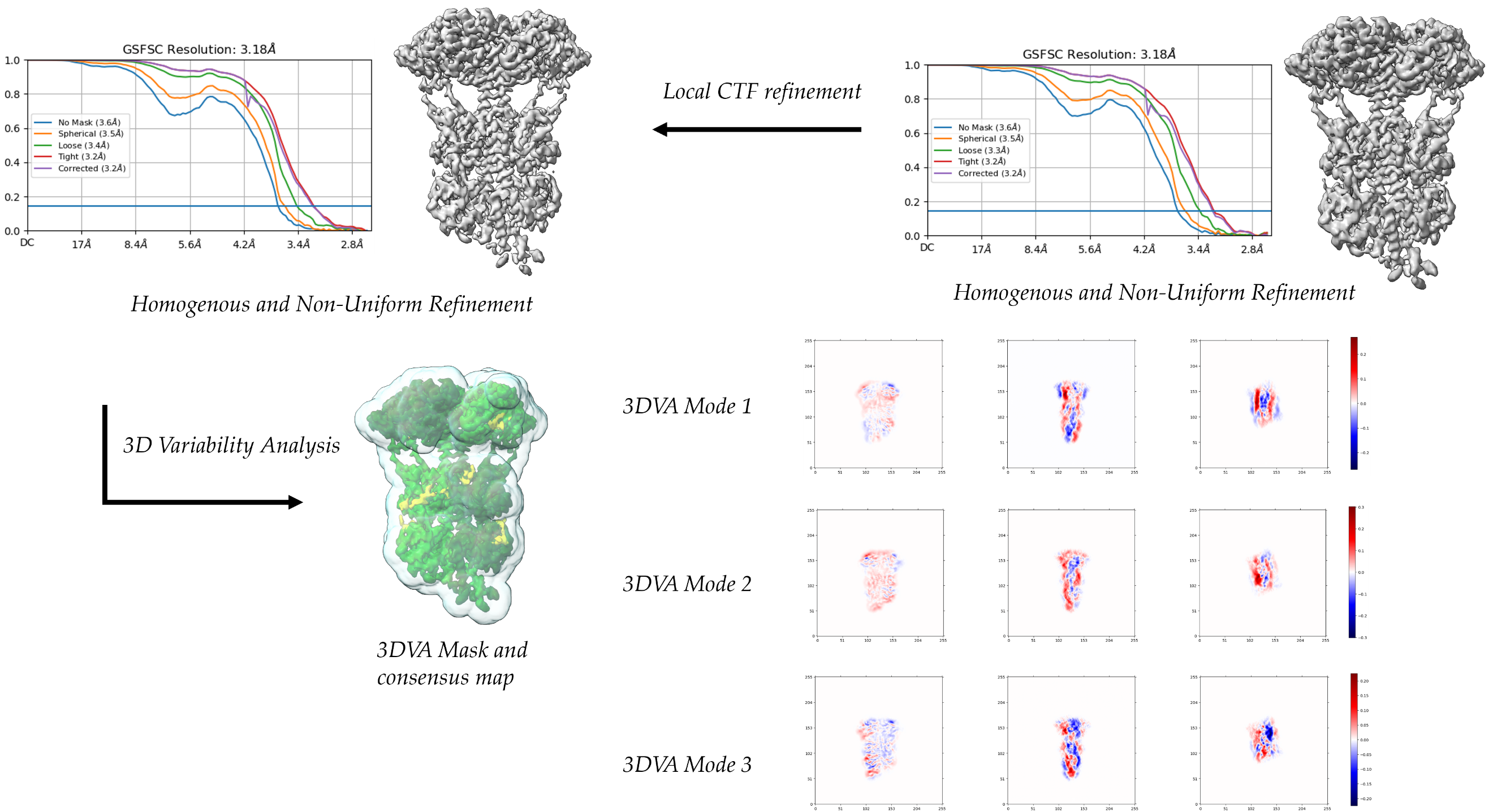


Figure S12: Cryo-EM data processing workflow for PDE6 bound to cGMP.


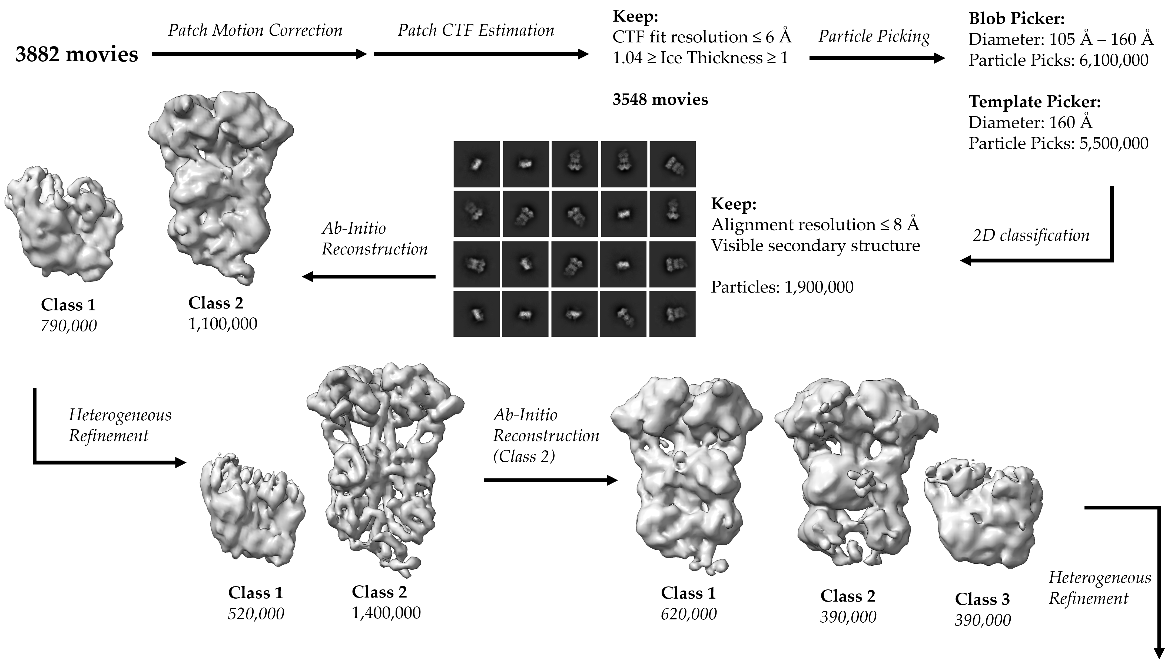

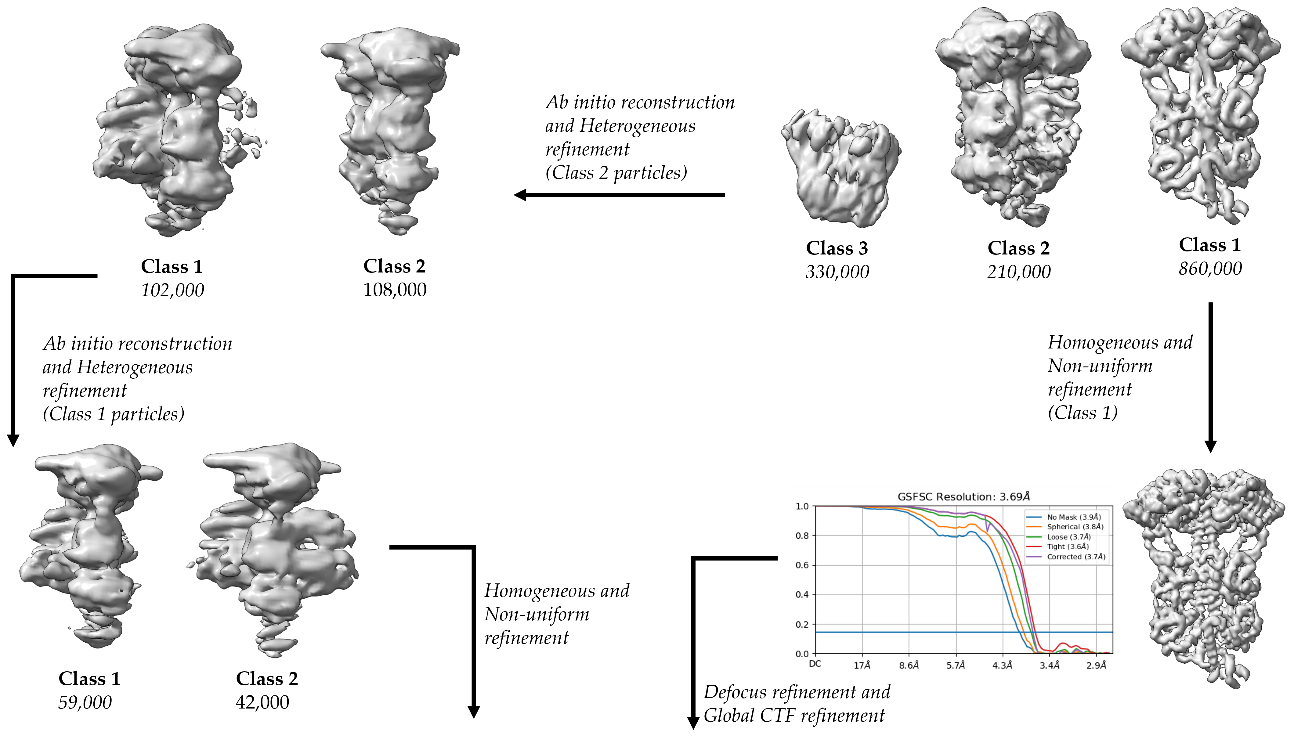

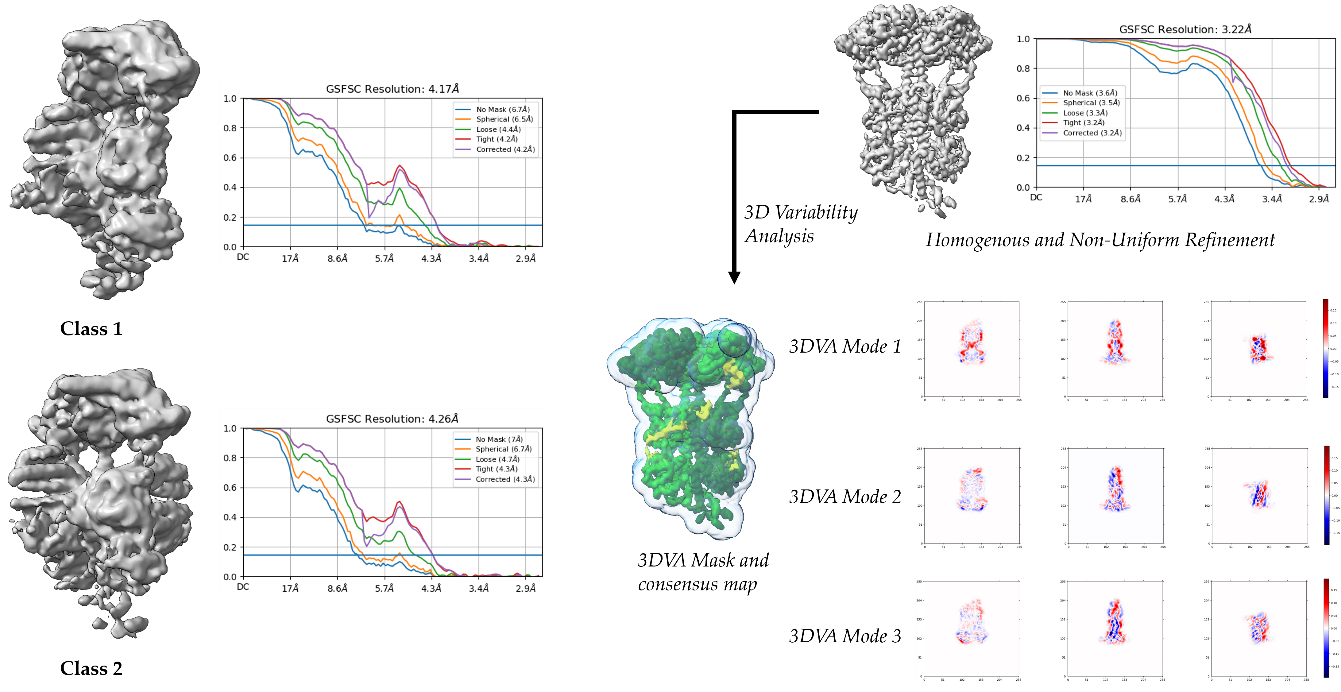


Figure S13: Cryo-EM data processing workflow for PDE6 bound to IBMX in the presence of retinal alpha.
